# Optogenetic Peripheral Nerve Immunogenicity

**DOI:** 10.1038/s41598-018-32075-0

**Published:** 2018-09-19

**Authors:** Benjamin E. Maimon, Maurizio Diaz, Emilie C. M. Revol, Alexis M. Schneider, Ben Leaker, Claudia E. Varela, Shriya Srinivasan, Matthew B. Weber, Hugh M. Herr

**Affiliations:** 1 MIT Media Lab, Center for Extreme Bionics, Massachusetts Institute of Technology, Cambridge, MA USA; 2 Harvard-MIT Division of Health Sciences and Technology (HST), Massachusetts Institute of Technology, Cambridge, MA USA; 3 Department of Electrical Engineering and Computer Science, Massachusetts Institute of Technology, Cambridge, MA USA; 4 Department of Bioengineering, École Polytechnique Fédérale de Lausanne (EPFL), Lausanne, Switzerland; 5 Department of Biological Engineering, Massachusetts Institute of Technology, Cambridge, MA USA; 6 Harvard-MIT Division of Health Sciences and Technology (HST), Harvard Medical School, Boston, MA USA

## Abstract

Optogenetic technologies have been the subject of great excitement within the scientific community for their ability to demystify complex neurophysiological pathways in the central (CNS) and peripheral nervous systems (PNS). The excitement surrounding optogenetics has also extended to the clinic with a trial for ChR2 in the treatment of retinitis pigmentosa currently underway and additional trials anticipated for the near future. In this work, we identify the cause of loss-of-expression in response to transdermal illumination of an optogenetically active peroneal nerve following an anterior compartment (AC) injection of AAV6-hSyn-ChR2(H134R) with and without a fluorescent reporter. Using Sprague Dawley Rag2^−/−^ rats and appropriate controls, we discover optogenetic loss-of-expression is chiefly elicited by ChR2-mediated immunogenicity in the spinal cord, resulting in both CNS motor neuron death and ipsilateral muscle atrophy in both low and high Adeno-Associated Virus (AAV) dosages. We further employ pharmacological immunosuppression using a slow-release tacrolimus pellet to demonstrate sustained transdermal optogenetic expression up to 12 weeks. These results suggest that all dosages of AAV-mediated optogenetic expression within the PNS may be unsafe. Clinical optogenetics for both PNS and CNS applications should take extreme caution when employing opsins to treat disease and may require concurrent immunosuppression. Future work in optogenetics should focus on designing opsins with lesser immunogenicity.

## Introduction

The clinical excitement surrounding optogenetics is eminently justified – a single, precise injection transforms a patient’s genome, making an anatomically targeted subset of neurons responsive to external control. Unlike electrical stimulation, the molecular specificity associated with unique promoters can limit off-target effects. Opsin engineering has conferred a toolbox of choices, each with its own favorable characteristics – photocurrent, wavelength, kinetics, and illumination sensitivity can be tuned to an application’s needs^1,2^. Lastly, an opsin’s DNA sequence is relatively short allowing it to be easily packaged within an AAV for stable delivery.

Using optogenetics to treat retinitis pigmentosa became the first clinical application, with chronic pain, cardiac pacing, and other PNS applications currently under investigation. Of currently available opsins, ChR2(H134R) was chosen for this study because it is one of the most commonly used opsins in the PNS^1^ and because it has optimal PNS properties including expression levels, kinetics, and photocurrent^3^. In March 2016, Retrosense Therapeutics (now Allergan) delivered the first in-human optogenetic therapy using AAV-ChR2 as part of an investigational new drug study. Gensight and Circuit Therapeutics are also working on optogenetic clinical models with potential in-human applications in the near future. These companies have largely been operating in the footsteps of Spark Therapeutics, which received FDA approval to market their RPE65 rAAV for retinal dystrophy in December 2017. Despite these rapid developments in rAAV and optogenetic deployment, academic research in the time-course, safety, and systemic tolerance of rAAV-administered optogenetic therapies within both PNS and CNS has lagged behind.

A limited number of previous studies describe optogenetic cytotoxicity and abnormalities; only a few discuss optogenetic expression time course in detail^4,5^. Axonal blebbing or puncta have been shown in response to both AAV and lentiviral delivered ChR2^6^ or NpHR^7^ under the αCaMKII promoter in the cortex *in vivo* and in acute brain slices from transgenic Thy1-NpHR mice^8^. While these abnormalities are associated with only a slight loss-of-function, they are directly attributed to high opsin production, endoplasmic reticulum (ER) retention, and a membrane trafficking defect. Other reports have focused on the opsin’s toxic effect on the cell membrane. High opsin expression has been shown to increase membrane capacitance^9^ and can lead to a loss of membrane integrity^10^.

In addition to cytotoxicity, phototoxicity has been shown in both retinal^11^ and cortical tissues^12^, where cellular damage is elicited by the high illumination intensities required to depolarize opsins. In an opsin-independent manner, the mechanism may be thermal^13^, although fluorescent reporters are also known to generate reactive oxygen species (ROS) directly in response to illumination; these ROS can elicit structural and DNA damage, and at high levels, initiate apoptosis^14^. Unlike phototoxicity, optogenetic excitotoxicity is directly related to light-induced opsin activation. The mechanism underlying excitotoxicity has been described as a process of undesirable intracellular acidification due to selective proton permeability^15^ and/or mitochondria-mediated apoptosis^16^. In the study proposing the latter, chronic blue light activation was employed to eliminate completely a resilient line of human melanoma cells selectively expressing ChR2(D156A)^16^.

Unlike ChR2 toxicity, AAV specific immunogenicity has been described in numerous studies. These studies range from the presence of anti-AAV neutralizing antibodies in human clinical trials to titer-specific inflammatory responses to AAV spinal cord microinjections in mice^1,17–19^ to anti-AAV antibodies in optogenetics^20^. Multiple strategies have been proposed to combat the immune response in AAV-mediated gene therapy including pharmacological intervention, dosing management, capsid decoys, and biomolecular engineering of AAV capsids^19,21^. In addition to cellular AAV responses, GFP cytotoxicity and immunogenicity have been described *in vivo* and *in vitro*^22,23^, although evidence suggests YFP is less cytotoxic than GFP^24^.

While two groups have studied immune responses specific to ChR2 within therapeutic optogenetics for blindness, neither group has reported significant immunogenic findings. Each group performs a biodistribution, an ocular toxicity study, and a systemic immunity study for either rAAV8-mGRM6-SV40-ChR2-heGFP or AAV2-CAG-ChR2-Venus intravitreal and/or subretinal injections^20,25^. Additional tests performed include complete blood counts (CBC), anti-AAV & anti-ChR2 serum antibody testing, and T cell population ratios. Neither study identifies any morphological or inflammatory changes in retinae using histology and GFAP, NF-κB, and CD45 immunohistochemistry. Systemic immunity studies revealed no toxicity in response to a delayed-type hypersensitivity (DTH) test and no abnormalities in CBC panels. Both studies conclude that AAV-ChR2 appears to be a safe method for restoration of vision in multiple mouse models of blindness.

While these results largely suggest AAV-ChR2 is safe for retinal transduction under specific promoter and injection conditions, several other analyses would be necessary to suggest more broadly the safety of AAV-ChR2 in mammals. For example, a DTH ear test without the mGRM6 regulatory sequence would allow one to conclude a low inflammatory response to the AAV-ChR2 construct as opposed to just the viral capsid itself. Further evaluation of the ChR2 DNA > 3X the threshold in muscle and liver may yield insights into whether the mechanism is viral clearance as opposed to the reported non-specific cross-contamination, especially given that liver and muscle are two of the highest concentration tropisms for AAV8 in intravenous delivery^26^, and that the liver is a key organ for adenovirus clearance^27^. Further studies of specific T-cell population targets could reveal additional insights regarding a potential role of a T-cell mediated immune response, given that systemic CBC levels may not change measurably in response to a small, targeted immune response in the eye. Lastly, a study of anti-ChR2 antibodies with a larger sample size and a more detailed time-course would yield stronger insights on the relationship of anti-transgene antibodies and expression levels. The above study introduced the possibility of a rat developing an anti-ChR2 antibody, but this only occurred in one rat, and not within the time-frame expected for antibody development (e.g., time-synced with either the elevated CD4+/CD8+ or CD4+/CD25+ T-cell ratios at 7 days or the elevated anti-AAV antibodies between 2 weeks and 2 months post-injection). To close these scientific gaps, additional studies are needed to address whether (a) therapeutic ChR2 is indeed immunogenic, (b) if so, this immunogenicity results in loss-of-expression, and (c) if so, the cause is the opsin (not AAV or reporter).

As the rat ages, the distance separating the surface of the skin and the nerve increases; in transdermal experiments, light scattering within tissue could prevent sufficient light fluence from reaching the target nerve resulting in the false appearance of a loss-of-expression^4^. Further, since loss-of-expression may be multifactorial, several of the above mechanisms may be co-contributing to the findings. For example, cytotoxicity may result in neuronal death, which could release some AAV capsid proteins and activate an adaptive immune response.

From the above studies, the precise mechanism behind optogenetic loss of expression over time remains unclear. Is the opsin downregulated or degraded over time in an intracellular process? Does this occur at the episomal DNA level, the mRNA level, or the protein level? Does high multiplicity of infection (MOI) and membrane blebbing lead to neuronal apoptosis? Does excessive illumination cause acidification-induced cell death? Is there an immune response? If so, is it humoral or cell-mediated, innate or adaptive? Is it directed against the AAV capsid, the opsin, or the fluorescent reporter?

To date, there have been no studies exhaustively analyzing the precise mechanism behind optogenetic peripheral nerve loss of expression. A mechanistic understanding *in vivo* could both confer enormous benefit to clinical trials that employ optogenetics to treat disease and enable previously unfeasible scientific studies that require stable, virally delivered optogenetics for a prolonged time-course. We summarize a list of mechanisms in Table 1. These mechanisms can be categorized within five groups: direct nerve damage, cytotoxicity, immunogenicity, protein downregulation, and anatomical changes. Of the above potential mechanisms, we hypothesize that ChR2 immunogenicity is the chief cause of the loss of optogenetic expression. Further, we hypothesize that the use of pharmacological immunosuppression can extend optogenetic expression longevity. We evaluate this hypothesis by comparing the optogenetic expression time-course of several AAV6 vectors with different promoters, with/without fluorescent reporters, and within wild-type (WT) rats, Rag2^−/−^ rats, and WT rats treated with tacrolimus, while performing bloodwork, histology, and gross anatomical observations to support the analysis.

**Table 1:** Comprehensive list of potential causes for loss of optogenetic expression over time.

General Mechanism	Specific Mechanism	Description	Likely Singular Physical Manifestation	Evidence in Literature
Direct Nerve Damage	Phototoxicity	Light-induced thermal damage	Axonal death + Wallerian Degeneration	Khan *et al*.^13^, Chen *et al*.^39^
Cytotoxicity	Excitotoxicity	Optogenetic pore-induced electrostatic damage	Neuronal death via apoptosis	Beppu *et al*.^15^,Perny *et al*.^16^, Lignani *et al*.^40^, Feldbauer *et al*.^41^
	ChR2 toxicity	Toxicity of opsin build-up & aggregation	Neuronal death via apoptosis	Zimmerman *et al*.^9^, Gradinaru *et al*.^10^, Miyashita *et al*.^6^, Li *et al*.^42^
	EYFP toxicity	Toxicity of reporter build-up & aggregation	Neuronal death via apoptosis	Ansari *et al*.^22^, Taghizadeh *et al*.^24^
Immunogenicity	AAV immunogenicity	Adaptive immune response to virus	Neuronal+ muscular death via CTL	Montgomery *et al*.^1^, Mingozzi *et al*.^19^, Sack *et al*.^43^, Kohro *et al*.^17^
	ChR2 immunogenicity	Adaptive immune response to opsin	Neuronal death via CTL	None^#^
	EYFP immunogenicity	Adaptive immune response to reporter	Neuronal death via CTL	Ansari *et al*.^22^, Stripecke *et al*.^23^ Kohro *et al*.^17^
Protein Loss-of-Expression	Episomal DNA loss	rAAV DNA broken down or lost during division	Neuron likely healthy, no longer expresses	McCarty *et al*.^44^, Fisher *et al*.^45^
	Epigenetic silencing	rAAV DNA silenced/methylated (no longer transcribed)	Neuron likely healthy, no longer expresses	Robertson *et al*.^46^, Okada *et al*.^47^, Migliaccio *et al*.^48^
	Transgene protein degradation or mRNA lysis/downregulation	Recombinant protein ubiquitinated and discarded via proteosome or no longer translated	Neuron likely healthy, no longer expresses	Jennings *et al*.^49^, Kong *et al*.^50^
Anatomical	Anatomical	More scattering in thick tissue	Neuron healthy, still expresses	Jacques *et al*.^51^, Maimon *et al*.^4^

^#^Non-opsin transgene immunogenicity has been seen in AAV gene therapy^19^ and has been discussed as a concern for optogenetics^52^.

## Results

### Optogenetic expression time-course not a function of dosage, rate age, or injection location

**Figure 1:**
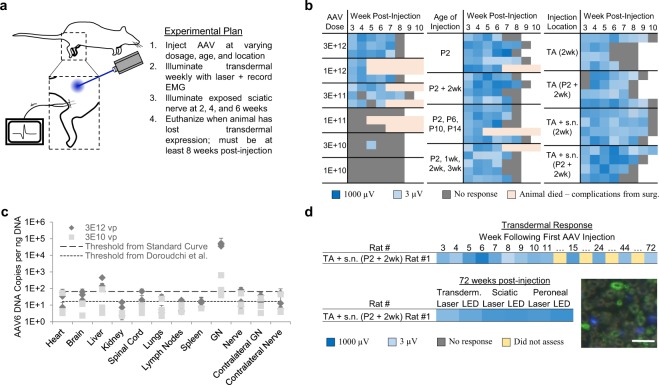
(**a**) Experimental plan for dosage, timing, and location of injection animals. (**b**) Logarithmic V_RMS_ amplitude of Tibialis Anterior (TA) motor activity in response to 473 nm, 45 mW/mm^2^ transdermal illumination of the proximal tibia for 4 s at 5 Hz and 10 ms PW for different groups. (2wk = 2 week, P2 = 2 day postpartum, s.n. = Sciatic Nerve) (**c**) Biodistribution results for 3E12 vp and 3E10 vp injected animals. (GN = Gastrocnemius Muscle, Nerve = Sciatic Nerve). n = 3 biologically independent samples for each group. (**d**) Time-course for Rat #1 in group TA + s.n. P2 & 2 wk. This animal did not lose transdermal expression up through 72 weeks post-injection, when rat was euthanized. Logarithmic V_RMS_ activity at the sciatic and peroneal nerves at time of euthanasia with different illumination sources shown, as well as sciatic nerve cross-section showing ChR2+ axons (green) and DAPI (blue): scale bar = 30 µm.

We have previously reported on the loss of transdermal optogenetic expression over time^4^. To address this issue, we first attempted to vary the injection dosage, the location of the injection, and the rat age during the injection to see if these variables affected the strength and time course of optogenetic activity levels (Fig. 1a). In line with the previous results, we found optogenetic activity levels to be heavily dose-dependent, with transdermal optogenetic activity beginning at dosages of 3E10 vp and increasing in strength through 3E12 vp (Fig. 1b). Injecting rats at different ages did not affect the time course of optogenetic activity; however, slightly weaker optogenetic activity was noticed in the rats injected over the first 4 weeks, likely due to synaptic pruning at the neuromuscular junction, which occurs rapidly in the first two weeks of murine development^28^. This is particularly apparent in the rats injected at 2 weeks as opposed to 2 days postpartum (P2). Additionally, loss of transdermal response appeared to occurr faster in the rats targeting the Tibialis Anterior (TA)-only as opposed to the TA & peroneal nerve at 2 weeks. However, no matter the dosing, timing or location of the injection, all of the rats with the exception of one had lost transdermal expression by 10 weeks post-injection (Fig. 1b). Amazingly, this one rat maintained a gradually weakening transdermal expression for up to 72 weeks post-injection, when it was euthanized for unrelated reasons (Supplemental Video, Fig. 1d). In the terminal procedure, this rat maintained strong levels of expression with direct stimulation of the sciatic and peroneal nerves with different illumination sources (Fig. 1d). However, despite this one animal, we could not sustain a long-term transdermal optogenetic response consistently with any specific dosing or injection scheme.

### Biodistribution suggests viral expression predominately in muscle tissue

The analysis of viral vector spread by quantitative PCR on DNA extract from various organs revealed only minor off-target dissemination (Fig. 1c). Viral DNA was found in high levels in the gastrocnemius (GN) muscle at the side of the injection for both concentrated and dilute injection groups, but in ~100x higher amounts for the concentrated injection group. Elevated levels in the liver were found to be consistent with standard bioclearance mechanisms^27^. Because the cervical spinal cord was sampled for biodistribution, and because AAV6 is likely to transverse retrograde only one synapse to the lumbar spinal cord, we expected, and identified no significant levels of rAAV DNA in the spinal cord. The sciatic nerve, targeted during the injection, also showed little to no viral DNA. This finding indicated that viral particles must travel in a retrograde fashion up the nerve via an intracellular pathway and that Schwann cells adjacent to each axon were not transduced by rAAV.

### Activated immune cells target ChR2-EYFP+ neurons in spinal cord

**Figure 2:**
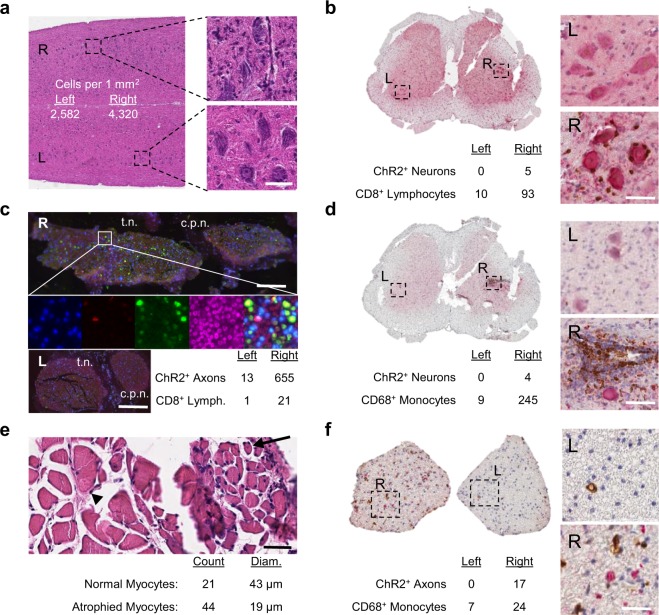
(**a**) Coronal lumbar H&E spinal cord section of ChR2-EYFP+ neurons in rat showing ipsilateral inflammation present. Scale bar = 80 μm. Experiment repeated 4 times with similar results. (**b**) Lumbar spinal cord cross-section immunohistochemistry for ChR2-EYFP (red), CD8 (brown), and hematoxylin (blue). Scale bar = 60 µm. Experiment repeated 3 times with similar results. (**c**) Sciatic nerve (R) and contralateral sciatic nerve (L) stained for ChR2-EYFP (green), CD8 (red), DAPI (blue), and background axons (magenta) with tibial (t.n.) and common peroneal (c.p.n.) nerve divisions labeled. Scale bar (R) = 250 µm; Scale bar (L) = 500 µm. Experiment repeated 2 times with similar results. (**d**) Lumbar spinal cord cross-section immunohistochemistry for ChR2-EYFP (red), CD68 (brown), and hematoxylin (blue). Scale bar = 60 µm. Experiment repeated 3 times with similar results. (**e**) Tibialis Anterior (TA) cross-section of ChR2-transduced rat showing myocytes which are healthy (arrowhead) and those with denervation atrophy (arrow): Scale bar = 60 µm. Experiment repeated 3 times with similar results. (f) Ventral root cross-section immunohistochemistry for ChR2-EYFP (red), CD68 (brown), and hematoxylin (blue). Scale bar = 20 µm. Experiment repeated 2 times with similar results.

We previously reported increased cell density within spinal cord samples of AAV6 transduced optogenetic rats as measured by DAPI+ fluorescence^4^. Here, we first evaluated H&E cross-sections of ChR2+ neurons in spinal cord samples; these cross-sections showed an ipsilateral inflammatory infiltrate predominately comprised of agranulocytes (Fig. 2a). We stained for CD8+ cytotoxic T lymphocytes (CTL) and discovered these CTLs appear to home to ChR2-EYFP transduced neurons directly (Fig. 2b); the ChR2+ neurons are surrounded by CTLs that otherwise are not present significantly within the gray matter of the spinal cord. To assess for further inflammation, we stained for CD68+ activated macrophages, and discovered an aggressive inflammatory infiltrate comprising many immune cells adjacent to the location of transduced ventral horn motor neurons (Fig. 2d).

We then explored sciatic nerve sections directly for evidence of immune cells (Fig. 2c). Although there was no significant evidence of increased cellularity, immunofluorescence for CD8+ lymphocytes revealed elevated CTLs scattered throughout the ipsilateral sciatic nerve expressing ChR2-EYFP, compared to only one CTL on the contralateral sciatic nerve. Within the injected muscle, there was distinctive evidence of denervation atrophy (Fig. 2e). Normal, healthy myocytes averaging 43 µm in diameter were found adjacent to distinctively shrunken myocytes averaging 19 µm in diameter. Comparatively, the contralateral TA did not show any evidence of shrunken myocytes (not shown). Despite the denervation atrophy, there was no evidence of inflammatory infiltrate within ipsilateral TA H&E sections, indicating that the muscle tissue itself was not an immune target. Lastly, ventral root sections showed increased presence of activated macrophages within roots containing ChR2-EYFP^+^ axons, as opposed to contralateral roots (Fig. 2f). Together, these results strongly suggested that ventral horn motor neurons and ChR2-EYFP transduced axons were being attacked by components of the adaptive immune system, resulting in motor neuron death and denervation atrophy in the corresponding muscle.

### ELISA shows ChR2-EYFP specific serum antibodies

Given the inflammatory infiltrate within spinal cord samples, it was theorized that the adaptive immune system was recognizing specific components of either the AAV6 capsid or the ChR2-EYFP fusion protein within the transduced neurons, which generally are co-localized. We developed an ELISA to identify rat serum antibodies against ChR2-EYFP protein, which showed a strong AAV injection dose dependency (Supp. Fig. 1b, 2c). The normalized antibody levels of the rats injected with 3E12 vp was significantly higher (P = 7E-4) at roughly 0.4 compared to 0.1 in the rats injected with 3E10 vp and 1E10 vp, suggesting the rats were developing an antibody response to the ChR2-EYFP fusion protein in a dose-dependent fashion.

Despite the high antibody titer, we could not yet conclude that adaptive immune response was actually causing a loss of optogenetic expression in the rats. After all, the ventral horn neurons being transduced by AAV6 are in the spinal cord, and the CNS is generally considered protected from immune responses via the blood-spinal cord barrier (BSCB). Further, the one animal that maintained transdermal optogenetic expression up to 72 weeks post-injection (Fig. 1d) was found to have elevated antibody levels as measured by a serum ELISA reading of 0.45, in line with other rats in his treatment group. If high antibody titer was directly causing loss-of-expression, we would expect this rat’s prolonged optogenetic activity to correlate with lower anti-ChR2-EYFP IgG levels. Lastly, we noticed that differences in the AAV6 injection timing or anatomical target had no significant effect on the overall ELISA antibody levels (P_ANOVA_ = 0.34), despite minor differences in optogenetic time-course within these groups, indicating that antibody levels did not perfectly correlate to loss of optogenetic activity (Supp. Fig. 1b). Together, these results suggested an immune response had occurred, but further experiments were required to identify conclusively if this immune response was causing loss-of-optogenetic expression or merely clearing dead neurons following cytotoxic or excitotoxic-mediated apoptosis.

### Rag2^−/−^ rats maintain optogenetic expression, implicating adaptive immunogenicity as key mechanism underlying loss of expression

**Figure 3:**
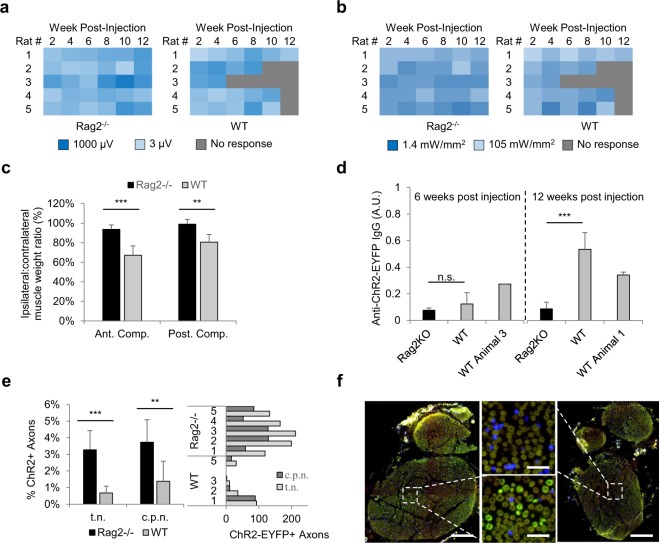
(**a**) Logarithmic V_RMS_ amplitude of Tibialis Anterior (TA) motor activity in response to 473 nm, 105 mW/mm^2^ transdermal illumination of the proximal tibia for 4 s at 5 Hz and 10 ms PW for Rag2^−/−^ and WT rats treated with high dose AAV6. (**b**) Logarithmic minimum transdermal illumination power needed to elicit transdermal EMG spikes from TA: V_RMS_ threshold set to 2.45 µV, which was empirically determined to be the max noise level of recordings. (**c**) Ipsilateral to contralateral side of injection muscle weight ratio at the time of euthanasia for high dose Rag2^−/−^ and WT rats in both the anterior and posterior compartment muscle groups, representing primarily the TA and Gastrocnemius muscles respectively (n = 5 per group). P = 1E-3 for Ant. Comp. and P = 3E-3 for Post. Comp. (**d**) Normalized ELISA comparing plasma antibodies against ChR2(H134R)-EYFP for Rag2KO and WT rats at 6 weeks post injection (n = 2 for Rag2KO, n = 5 for WT) and 12 weeks post injection (n = 5 for both groups). P = 8E-4 for 12 week. WT Animal 3 was the only rat which lost transdermal optogenetic expression at week 6. WT Animal 1 was the only rat which maintained expression at week 12. In addition to being included in their respective groups, these animals are also shown separately. (**e**) ChR2-EYFP+ axon counts as percentage of total axons (left) and as absolutes (right) in tibial nerve (t.n.) and peroneal nerve (c.p.n.) divisions of sciatic nerve of WT and Rag2^−/−^ rats (n_Rag2−/−_ = 8, n_WT_ = 7): P_left_ = 2E-4. P_right_ = 3E-3. On left, rats from excitotoxicity control group also included. WT Rat #4 sciatic nerve omitted because was not properly paraffin processed. (**f**) Sciatic nerve cross sections of representative Rag2^−/−^ rat (left) and WT rat (right) labeled for ChR2-EYFP (green) and DAPI (blue). Scale bar_left,right_ = 120 µm, Scale bar_center_ = 20 µm. Experiment repeated 3 times with similar results.

To identify causality, Rag2^−/−^ rats were employed. These rats were first phenotypically assessed to verify adaptive immune deficiency. Tail vein blood from WT and Rag2^−/−^ Sprague Dawley rats at 6 weeks of age showed a significant difference in total white blood counts (WBC), driven predominately by lymphocyte deficiency in the Rag2^−/−^ group (Supp. Fig. 2a). Since Rag2 deficiencies do not affect innate immune lymphocyte populations, we employed flow cytometry to determine the remaining Rag2^−/−^ lymphocytes were CD3^−^, suggesting the lack of mature T cells (Supp. Fig. 2b). We then injected AAV6 as before to transduce ventral motor neurons of Rag2^−/−^ and WT rats and found that at 4 weeks post-injection, both groups had similar levels of optogenetic activation. EMG responses were present in 5/5 rats of similar magnitude (P = 0.31). However, by 12 weeks post-injection, 4/5 of the WT rats had lost all optogenetic responses to transdermal illumination compared to 0/5 of the Rag2^−/−^ rats (Fig. 3a). Further, the minimum illumination power required to activate the targeted peroneal nerve stayed relatively constant in the Rag2^−/−^ rats, as compared to the WT rats, which stopped responding, even at maximum illumination power (Fig. 3b). This effectively ruled out anatomical changes alone, because the fluence change was not sufficient to prevent transdermal optogenetic activation of the nerve for Rag2^−/−^ rats, the males of which were much larger than their WT female counterparts, all of whom had stopped responding to transdermal illumination. The EMG results correlated well with the immunofluorescence findings which showed significantly greater counts of ChR2-EYFP+ axons in the sciatic nerves of Rag2^−/−^ rats compared to the WT rats (Fig. 3e,f), as well as CD8+ lymphocytic inflammation only in the spinal cords of WT rats (Supp. Fig. 2d,e).

In agreement with the loss-of-expression findings, ipsilateral muscle atrophy was also found to be restricted to the WT rats (Fig. 3c). Ipsilateral muscles were 33 ± 9% reduced and 19 ± 8% reduced compared to contralateral muscle weights in the anterior and posterior compartments respectively for WT rats. Comparatively, Rag2^−/−^ rats had muscle reductions of 6 ± 5% and 1 ± 5% for the same compartments respectively. Anterior compartment muscle mass reductions ranged from a high of 47% for WT Rat #3 to a low of 23% for WT Rat #1, which correlates precisely with their optogenetic expression profiles. WT Rat #3 was the first rat to lose expression and therefore may have had the strongest immune response; WT Rat #1 had yet to lose expression at the time of euthanasia and thus may have had the weakest immune response. These individual differences were also reflected in the ChR2-EYFP ELISA. At 6 weeks post-injection, the plasma antibodies against ChR2-EYFP were slightly larger in the WT group, compared to the Rag2^−/−^ group (P = 0.29) (Fig. 3d). However, the highly immunogenic WT Rat #3 was the outlier, with more than 2X the WT group’s overall ChR2-EYFP specific plasma antibody levels at that time. By 12 weeks post-injection, the serum antibodies in the WT group increased significantly, reaching 6X the Rag2^−/−^ group (P = 8E-4). At this time, WT Rat #1 was the outlier with roughly half of the antibody expression of the group as a whole, in line with the lower atrophy and maintained immunofluorescent axonal expression in this rat (Supp. Figure 2f).

To rule out excitotoxicity as a contributing factor to loss of expression, we found the WT excitotoxicity controls showed similar results to the WT stimulated rats during terminal procedures. 4/4 WT rats showed no response to any transdermal illumination during terminal procedures compared to 0/4 of the Rag2^−/−^ excitotoxicity controls (Supp. Fig. 6a). Further, no significant muscle atrophy, nerve expression, or ELISA differences were found between stimulation and no-stimulation groups (Supp. Fig. 6b,d) as compared to the sigificant differences in muscle atrophy, nerve expression, and antibody levels between Rag2^-/-^ and WT no-stimulation groups (Supp. Fig. 6b-d). This result suggests that the stimulation used in this experiment is insufficient to cause any excitotoxicity mediated nerve damage or neuronal death. Together with the stimulation results, these data show conclusively that the adaptive immune system is a necessary condition for the loss of optogenetic activation over time and that a component of the AAV6-ChR2-EYFP immunogen is directly causing loss of expression.

### Tacrolimus extends longevity of optogenetic expression in WT rats

**Figure 4:**
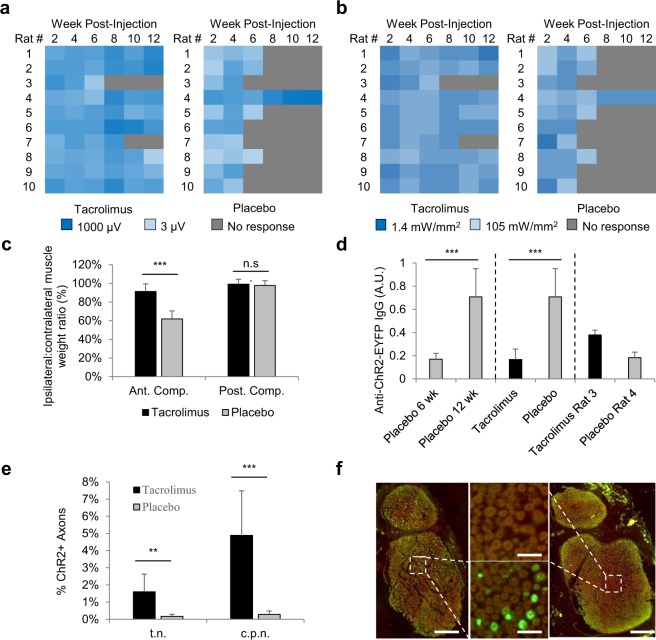
(**a**) Logarithmic V_RMS_ amplitude of Tibialis Anterior (TA) motor activity in response to 473 nm, 105 mW/mm^2^ transdermal illumination of the proximal tibia for 4 s at 5 Hz and 10 ms PW for rats treated with slow release tacrolimus pellet compared to rats treated with placebo pellet. (**b**) Logarithmic minimum transdermal illumination power needed to elicit transdermal EMG spikes from TA for tacrolimus-treated and placebo-treated rats: V_RMS_ spike threshold set to 2.45 µV, which was empirically determined to be the max noise level of recordings. (**c**) Ipsilateral to contralateral side of injection muscle weight ratio at the time of euthanasia between tacrolimus and placebo rats for both the anterior and posterior compartment muscle groups, representing primarily the TA and Gastrocnemius muscles respectively (n = 8 for tacrolimus, 9 for placebo group). Tacrolimus rats #3 and #7 (which had lost expression at time of euthanasia), and placebo rat #4 (which maintained expression at time of euthanasia) were excluded, as shown in Supplemental Figures. P = 3E-6 for anterior compartment and P = 0.23 for posterior compartment. (**d**) Normalized ELISA comparing plasma antibodies against ChR2(H134R)-EYFP for WT rats at 6 weeks post injection (n = 10) and 12 weeks post injection (all others, n = 10). P = 5E-5 for 6 vs 12 week comparison. P = 3E-5 for tacrolimus vs. placebo comparison at 12 weeks. In addition to being included in their respective groups, rat #3 in tacrolimus group and rat #4 in placebo group are also shown separately as they are outliers in their respective groups. (**e**) ChR2-EYFP+ axon counts as percentage of total axons in tibial nerve (t.n.) and common peroneal nerve (c.p.n.) of tacrolimus and placebo rats (n_Tacrolimus_ = 8, n_Placebo_ = 9): P_t.n._ = 5E-3; P_c.p.n._ = 1E-3. Tacrolimus rats #3 and #7 (which had lost expression at time of euthanasia), and placebo rat #4 (which maintained expression at time of euthanasia) were excluded. (**f**) Sciatic nerve cross sections of representative Rag2^−/−^ rat (left) and WT rat (right) labeled for ChR2-EYFP (green) and DAPI (blue). Scale bar_left,right_ = 120 µm, Scale bar_center_ = 20 µm. Experiment repeated 7 times with similar results.

To identify pharmacological candidates for extending the life of virally delivered optogenetic expression in a WT animal, two broad categories of FDA-approved drugs were evaluated: a monoclonal antibody against CD49d (PS2), also known as VLA-4 or α4 integrin, and a general immunosuppressant. As the murine analog of natalizumab, a treatment for Multiple Sclerosis (MS), PS2 was chosen for its anti-inflammatory effects in experimental autoimmune encephalomyelitis (EAE)^29^. Compared to the placebo, PS2 was not found to increase the longevity of optogenetic activation, with 10/10 PS2 rats and 9/10 placebo rats losing all responsiveness to transdermal illumination by week 8 (Supp. Fig. 4a). Comparatively, the general immunosuppressant tacrolimus was found to increase signfiicantly the longevity of transdermal optogenetic expression with only 1/10 tacrolimus treated rats losing optogenetic responsiveness to transdermal illumination at week 8, compared to 9/10 placebo rats (Fig. 4a). Additionally, PS2 did not appear to have any effect on WBC counts (data not shown) as compared to tacrolimus, which significantly reduced monocyte and lymphocyte counts compared to placebo (Supp. Fig. 3a). Further, PS2 rats were found to have developed non-specific anti-antibody neutralizing antibodies, which may have precluded drug efficacy (Supp. Fig. 4c).

Similar to the Rag2^−/−^ rats, all rats treated with tacrolimus or placebo responded well to transdermal illumination at 4 weeks post-injection (Fig. 4a). However, at 6 weeks, only 5/10 of the placebo rats responded to transdermal illumination compared to 10/10 of the tacrolimus rats. By 8 weeks, only 1/10 placebo compared to 9/10 tacrolimus rats responded with muscle activity to transdermal illumination. This one outlier placebo rat continued to increase in expression strength until 12 weeks, whereas two of the tacrolimus rats (#3, #7) lost transdermal expression by 12 weeks. The minimum fluence rate to elicit electrophysiological spikes similarly showed increasing sensitivity of placebo rat #4 and maintained sensitivities of all tacrolimus rats except #3 and #7 (Fig. 4b), which aligns precisely with the low ChR2+ axon counts in all placebo rats except #4 and high ChR2+ axon counts in all tacrolimus rats except #3 and #7 (Supp. Fig. 3b,e).

As with the WT rats, there was significant muscle atrophy within the anterior compartment of the placebo group of 38% as compared to 9% in the tacrolimus group (P = 3E-6) (Fig. 4c). The atrophy ranged from a high of 57% to a low of 28% for the placebo rats and a high of 23% to a low of −6% for the tacrolimus rats, when omitting the three outliers: tacrolimus rats #3 and #7 & placebo rat #4, which had atrophy of 58%, 43%, and 6% respectively (Supp. Fig. 3c). Unlike the WT rats, the posterior compartments in both tacrolimus and placebo groups did not show any atrophy at all (3% and 1%), possibly indicating better targeting of the anterior compartment during the injection. Similar to the WT rats, the ELISA results in the placebo group support the findings of ChR2-EYFP immune responses (Fig. 4d). At 6 weeks, the measured anti ChR2-EYFP plasma antibodies of the placebo group is small, increasing significantly by 12 weeks (P = 5E-5). At 12 weeks, the tacrolimus group maintained low plasma antibodies as compared to the placebo group (P = 3E-5) with tacrolimus rat #3 and placebo rat #4 as exceptions, in line with muscle atrophy and loss-of-expression profiles. These data show that tacrolimus can be used to increase significantly the length of optogenetic expression.

It is unclear exactly what precipitated the lack of immune response for outlier placebo rat #4. Of note, at 6 weeks post-injection, this rat was found to be very anemic, with red blood cells (RBC), hemoglobin (Hb), and hematocrit (HCT) levels all 8 standard deviations below the average from the other 9 placebo animals (Supp. Fig. 3d). Interestingly, the only other rat which maintained expression at week 12 (WT rat #1) was also anemic at 6 weeks. This rat’s RBC, Hb, and HCT levels were found to be 5 standard deviations below the other 4 WT rats in its group (Supp. Fig. 3d). However, the white counts (including differentials) and platelet counts for these two outliers were not significantly different from counts of their respective groups (data not shown). In addition, we noticed that tacrolimus had an effect on hematological characteristics. Within the tacrolimus group, RDW was significantly elevated (P = 1E-3) along with a corresponding reduction in MCV (P = 9E-4) and MCH (P = 4E-3) compared to the placebo group (Supp. Fig. 3f). However, no significant anemia was present between the placebo and tacrolimus groups (P_Hct,Hb_ = 0.7), even when excluding outlier placebo rat #4. We can therefore conclude that tacrolimus is causing a reduction in the average erythrocyte size. Since this is occurring when tacrolimus concentration hits peak levels around 4 weeks of age, and knowing that the rat erythrocyte life is roughly 60 days^30^, we can conclude that the high RDW and low MCV may be explained by the combination of normal erythrocytes prior to and small erythrocytes following the administration of tacrolimus therapy.

### CAG promoter rules out AAV capsid as primary cause of immune response

**Figure 5:**
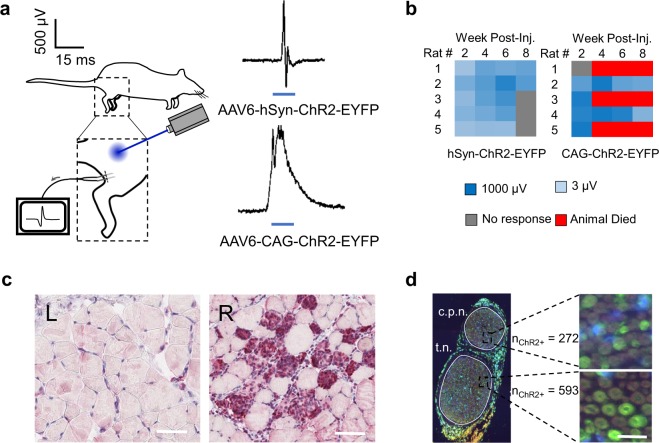
(**a**) Tibialis Anterior (TA) EMG recordings in response to 473 nm transdermal illumination of proximal tibia in rats transduced with rAAV-ChR2 restricted by either hSyn or CAG promoter. (**b**) Logarithmic minimum transdermal illumination power needed to elicit transdermal EMG spikes from TA for hSyn and CAG promoters: V_RMS_ spike threshold set to 2.45 µV, which was empirically determined to be the max noise level of recordings. (**c**) TA cross-section of CAG rat 8 weeks post-injection showing healthy myocytes devoid of inflammation on contralateral limb (left) and ChR2-EYFP transduced myocytes co-localized with significant inflammatory cells on ipsilateral limb (red, right): scale bar = 40 µm. Experiment repeated 1 time with similar results. (**d**) Immunofluorescence for ChR2+ axons in CAG-ChR2-EYFP sciatic nerve sections along with counts in peroneal (c.p.n.) and tibial (t.n.) sections: scale bar = 20 µm. Experiment repeated 1 time with similar results.

To identify the role of AAV capsid in precipitating an immune response, we evaluated the ChR2-EYFP injected under the CAG promoter. Unlike hSyn, CAG does not restrict ChR2 expression to neural tissue, which was shown by the unique spiking behavior following transdermal illumination of these rats (Fig. 5a). Whereas hSyn spikes are narrow and return to baseline within ~4 ms of activation, CAG spikes are wide and return to baseline ~20 ms following activation. The narrow coordinated spike at the beginning of an optogenetically activated CAG waveform represents direct neural firing – all myocytes are time-synced to depolarize at once. This is followed by wider-band activity from continual activation of the myocytes directly, which have different refractory dynamics as compared to axonal activation. This distinction is in agreement with our previous findings showing no motor activity following direct illumination of muscle in hSyn rats^4^ as compared to CAG rats, which showed exquisite motor control as would be expected with direct myocyte activation (Supplemental Video).

Unlike the hSyn animals, the CAG rats exhibited excessive mortality of 60% (Fig. 5b). Although necropsies did not reveal any specific cause of death, these animals overall had a lower weight than the WT rats and exhibited more hunching and eye staining. Like the hSyn promoter the two surviving CAG rats lost strength of the EMG signal from week 4 to week 8 in terms of EMG levels. Unlike the hSyn promoter, an immunohistochemical analysis of the CAG TA showed significant ChR2+ myocytes. Not only were these myocytes spread throughout the section, there was lymphocytic inflammation specifically co-localized to the transduced myocytes (Fig. 5c). The biodistribution for CAG rats revealed levels of ChR2-EYFP DNA within both the CAG and hSyn muscle were very similar at ~1E5 copies per ng DNA (Supp. Fig. 5A). Therefore, given the identical AAV6 dosage and injection, and similar biodistribution results between the hSyn and CAG groups, the findings of high mortality and ChR2-EYFP specific myocyte inflammation in exclusively the CAG rats must indicate that the immune response is directly targeting ChR2-EYFP and not the AAV capsid. Since both hSyn and CAG promoters result in equivalent levels of AAV capsid fragment expression on myocytic MHCI, but only the CAG promoter results in myocytic immune attack, we can conclude immunogenicity is specific to the  ChR2-EYFP protein.

### ChR2-only and ChR2-EYFP rats show equivalent immune activity, suggesting immune response is ChR2-specific

**Figure 6:**
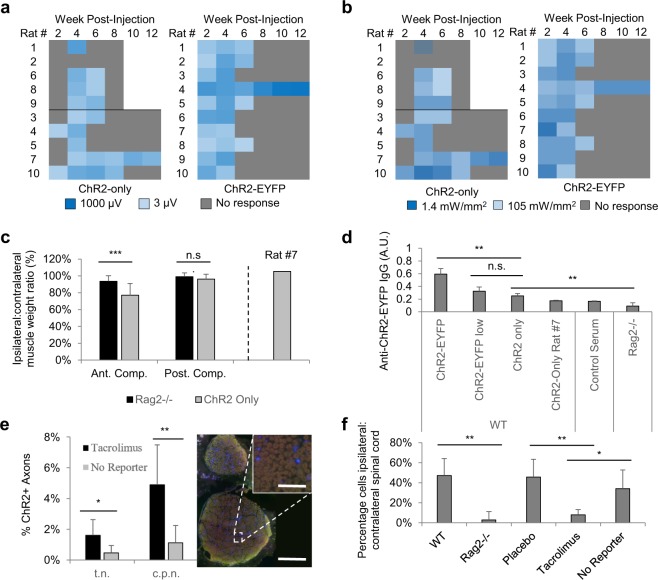
(**a**) Logarithmic V_RMS_ amplitude of Tibialis Anterior (TA) motor activity in response to 473 nm, 105 mW/mm^2^ transdermal illumination of the proximal tibia for 4 s at 5 Hz and 10 ms PW for rats injected with ChR2-only vs. those with ChR2-EYFP. (**b**) Logarithmic minimum transdermal illumination power needed to elicit transdermal EMG spikes from TA for ChR2-only and ChR2-EYFP rats: V_RMS_ spike threshold set to 2.45 µV, which was empirically determined to be the max noise level of recordings. (**c**) Ipsilateral to contralateral side of injection muscle weight ratio at the time of euthanasia between Rag2^−/−^ and ChR2-only rats for both the anterior (A.C.) and posterior compartment (P.C.) muscle groups, representing primarily the TA and Gastrocnemius muscles respectively (n = 9 for Rag2^−/−^, n = 9 for ChR2 only). ChR2-only rat #7 (which had maintained expression at time of euthanasia) was also shown separately and excluded from significance test (P_A.C._ = 7E-5, P_P.C._ = 0.06). (**d**) Normalized ELISA comparing plasma antibodies against ChR2(H134R)-EYFP for WT rats at normal and low dose, Rag2^−/−^ rats at normal dose, and ChR2(H134R) WT rats without reporter (n_ChR2-EYFP_ = 4, n_ChR2-EYFP low_ = 4, n_ChR2 only_ = 3, n_Rag2_^−/−^ = 5). Only ChR2-only rats who had lost all transdermal and subcutaneous expression at the time of blood collection were included. P_left_ = 0.002; P_center_ = 0.12; P_right_ = 0.006. (**e**) ChR2-EYFP+ axon counts as percentage of total axons in tibial nerve (t.n.) and peroneal nerve (c.p.n.) of tacrolimus and no reporter rats (n_Tacrolimus_ = 8, n_NoReporter_ = 9): P_t.n._ = 0.02; P_c.p.n._ = 4E-3. Tacrolimus rats #3 and #7 (which had lost expression at time of euthanasia), and no reporter rat #7 (which maintained expression at time of euthanasia) were excluded. Sciatic nerve cross sections of representative no reporter rat labeled for ChR2 (green) and DAPI (blue). Scale bar_bottom_ = 200 µm, Scale bar_top_ = 30 µm. (**f**) Number of DAPI+ cells on ipsilateral side compared to contralateral side of spinal cord expressed as a percentage increase for WT (n = 3), Rag2^−/−^ (n = 3), placebo (n = 3), tacrolimus (n = 3), and no reporter rats (n = 5). P_ANOVA_ = 8E-3. Can reject the null using Fisher’s LSD at α = 0.01 for ^** ^and α = 0.05 for ^*^.

After determining the primary immunogen is ChR2-EYFP, we injected a last set of 10 rats with AAV6-hSyn-ChR2 lacking the fluorescent reporter. Interestingly, these animals at 2 and 4 weeks expressed at significantly weaker levels than those containing the fluorescent reporter (P = 0.05) (Fig. 6a). Further, the movement was qualitatively weaker (Supplemental Video), although the minimum fluence rates needed to activate the nerves at 4 weeks was similar (Fig. 6b). Because of the above, it is likely that fewer axons expressed functional ChR2 at 4 weeks of age in absence of reporter, although 12 week ChR2+ axon counts were insignificantly different (Fig. 6e, Supp. Fig. 7a). Without reporters, fluorescent activity for all sciatic nerves ranged between 0–2% of total axons with the exception of rats #2 and #7, which had peroneal transduction rates of 4% and 9% of all axons respectively, which matches electrophysiological findings. Additionally, Rat #7 still expressed at the time of euthanasia, optogenetically responding to transdermal stimulation. The general finding of weaker optogenetic activation in absence of reporter is in agreement with previously unpublished reports by other groups that the presence of the reporter itself increases the photocurrents of the opsin molecule, although the mechanism underlying this remains unclear.

As with the previous WT rats, ChR2-only rats revealed ipsilateral muscle loss in the anterior compartment of 23 ± 14% (Fig. 6c). Among the rats that lost expression in this group, muscle weight loss ranged from 35% in rat #1 & #10 to −5% in Rat #7, likely reflecting a drop in the number of optogenetically active motor neurons compared to the WT and placebo groups of same dosage. To verify this AAV did not contain EYFP, immunofluorescence using antibodies against EYFP revealed no axons, whereas using antibodies against ChR2 yielded transduced axons (Supp. Fig. 7d). To verify immunogenicity as opposed to toxicity in the ChR2-only group, immunohistochemistry of axial spinal cord sections revealed both a high number of CD8+ lymphocytes on the injection side as compared to the contralateral side (Supp. Fig. 1b) and generally high cell densities on the ipsilateral side (Supp. Fig. 7c). Further, the ELISA against ChR2 revealed a slight elevation of anti-ChR2 serum antibodies in the no reporter group as compared to the Rag2^−/−^ rats and non-injected control samples; however, the serum antibody level was significantly lower than those of the same dose ChR2-EYFP injections (Fig. 6d). Levels were insignificantly different from the antibody levels of the low-dose ChR2-EYFP injections in the WT rats. No reporter rat #7, which maintained expression at the time of euthanasia, had lower levels of plasma antibodies, in line with the non-injected sample. When high cell densities in the spinal cord were evaluated as a percentage increase over the contralateral side, placebo, WT, and no reporter rats all have significant ipsilateral inflammation compared to Rag2^−/−^ and Tacrolimus treated groups: P_ANOVA_ = 8E-3 (Fig. 6f). Together, these results definitively implicate the ChR2 transgene protein as being the highly immunogenic cause of optogenetic loss-of-expression. In this experiment, the ChR2 transgene elicited a strong CTL-mediated PNS and CNS immune response resulting in loss of optogenetic activation, motor neuron death, and muscle atrophy.

The contribution of each experiment to the conclusive establishment of the immunogenic cause is outlined in Table 2. Although other factors are not conclusively ruled out as contributing to loss-of-expression, the results show that ChR2 immunogenicity is definitively causing loss of expression and motor neuron death; it is a prime concern for optogenetic time-course.

**Table 2:** Contribution of factors to conclusion of ChR2-specific immunogenicity.

Potential Causes	TA histology	Spinal Cord IHC	Sciatic IF	Rag2^−/−^ Electrophys.	Excitotox. Control	Immuno-suppression	CAG muscle histology	CAG mortality	No Reporter
Phototoxicity	**+**		−						
Excitotoxicity	**+**				−				
ChR2 toxicity	**+**			−					
EYFP toxicity	**+**			−					
AAV immunogenicity	**+**	**+**	**+**	**+**		**+**	−	−	
ChR2 immunogenicity	**+**	**+**	**+**	**+**		**+**	+		**+**
EYFP immunogenicity	**+**	**+**	**+**	**+**		**+**	+		−
Episomal DNA loss	−	−							
Epigenetic silencing	−	−							
Transgene protein degradation or mRNA lysis	−	−							
Anatomical	−	−		−					
Rationale	Denervation atrophy suggests neuronal damage	CD8+ & CD68+ WBC home to ChR2+ Neurons	Axons healthy, with slight elevation of CTLs	5/5 Rag2^−/−^ compared to 1/5 WT with expression	WT rats low expression & pos. immune panel whether stim or not	Tacrolimus rats maintain expression & neg. immune panel	Inflammation only present in CAG-ChR2-EYFP+ myocytes	AAV is same, but significant mortality in CAG rats	Loss of expression & pos. immune panel without reporter

## Discussion

In this experiment, we hypothesize that ChR2 immunogenicity is causing the loss-of-expression over time in Sprague Dawley and Fischer 344 rats. The data presented in this study support this hypothesis. To identify immunogenicity, we employ Rag2^−/−^ rats and identify sustained levels of transdermal optogenetic expression at 12 weeks post-injection. Further, we use a slow-release tacrolimus to extend the time course of expression levels in a WT rat. We then study AAV-hSyn-ChR2 upon removal of the fluorescent reporter, and identify all the same signs of immunogenicity, suggesting ChR2 alone is still highly immunogenic.

Unlike previous studies, which have shown loss-of-optogenetic activity may be caused by AAV immunogenicity, EYFP toxicity, ChR2 excitoxicity, or other potential mechanisms, this is the first study to show that ChR2 immunogenicity is directly causing loss-of-optogenetic activity in the CNS/PNS, neuronal death, and muscle atrophy. As a result, we raise some concerns regarding the application of ChR2 as a therapeutic tool. Perhaps optogenetic therapies should only be applied for CNS neurons that do not project into the PNS? Perhaps once foreign proteins in the CNS are recognized by the immune system, the Blood Brain Barrier (BBB) or BSCB become more permeable to immune cell passage? Perhaps early clinical translations of optogenetic therapies should recruit patients who are already immunocompromised instead of screening out those patients? Future scientific work and clinical work should aim to address these questions.

We develop a comprehensive panel of four tests that identify an optogenetic immune response. First, a novel ELISA identifies serum antibodies against ChR2 or ChR2-EYFP. We validate this ELISA using serum from an AAV-ChR2 dosage curve. The ELISA properly identifies group outliers, suggesting it may be a method of predicting loss-of-optogenetic-activity. Second, ipsilateral muscle atrophy as assessed by muscle weight is correlated with loss of expression. In the future, using muscle mass reduction to provide an estimate of transduction efficiency may be more accurate than using nerve cross-sections, because nerve cross-sections may be biased by variability in branching of cutaneous, autonomic, or non-target muscle efferent nerve fibers. Third, immunofluorescent observations of nerve fibers and spinal cord sections reveal elevated CD8+ lymphocytes and CD68+ macrophages co-localized to ChR2+ neurons and axons. Lastly, DAPI+ cell counts of contralateral and ipsilateral spinal cord sections show an increase of ipsilateral inflammatory cell density. Together, these four tests comprehensively constitute a peripheral optogenetic-specific immune panel, which can be employed as a benchmark in future scientific and clinical optogenetic studies.

We present thirteen potential mechanisms describing loss-of-optogenetic activity divided into five independent categories by mechanism of action: phototoxicity, cytotoxicity, immunogenicity, protein downregulation, and anatomy. While each of these mechanisms may play some role in loss-of-activity, our experiments suggest that ChR2 immunogenicity is the key contributor. To reach this conclusion, we first identify inflammation in spinal cord samples in certain WT rats. However, cellular apoptosis can also send damage signals and recruit inflammatory cells – spinal cord inflammation is not alone sufficient to rule out phototoxic, excitotoxic or cytotoxic mechanisms. The presence of CTLs and activated macrophages co-localized to ChR2+ neurons strongly suggests an adaptive immune process is occurring directly related to the AAV capsid or viral transgene. However, to prove this causatively, we employ Rag2^−/−^ rats and find extended transdermal optogenetic activity in all Rag2^−/−^ rats. We attempt to prolong optogenetic expression in WT rats and find that a subcutaneous slow-release tacrolimus pellet is an effective method to extend optogenetic expression. To rule out AAV capsid immunogenicity, we inject two WT groups with the identical AAV dose, using CAG and hSyn promoters to modulate tissue specificity. Not only does the CAG promoter result in significant mortality, but it also results in immune infiltrates co-localized with ChR2+ myocytes. Immune infiltrates do not occur within the ChR2- hSyn promoter myocytes, despite the identical presence of AAV capsid within muscle tissue as shown by the biodistribution, enabling us to conclude that the adaptive immune system is targeting the ChR2-EYFP protein specifically. Lastly, we perform AAV-ChR2 injections without the EYFP reporter. We discover the same loss-of-expression time-course as with the EYFP reporter, allowing us to conclude that of the thirteen potential mechanisms, ChR2 immunogenicity is the chief cause.

In addition, we present outliers to the groups revealing unique insights for further study. One rat out of the fifty-four studied in the dosing, timing, and anatomy study maintained transdermal peripheral nerve expression for 72 weeks post-injection. This represents the longest-ever virally induced peripheral nerve optogenetic response to our knowledge, indicating that highly expressing long-term optogenetic stability is possible, even in the case of elevated levels of plasma anti-ChR2-EYFP antibodies. To understand why this might have been the case, we analyze hematological abnormalities of WT rat #1 and placebo rat #4, both of which maintain transdermal expression when the other rats in their respective groups lose expression by week 12. It is interesting to note that both of these rats were significantly anemic at 6 weeks of age compared to other rats in their groups (P: ~1E-18 and ~1E-7), despite no difference in WBC or thrombocyte counts. At this age, only a few rats had lost transdermal optogenetic expression, so the early anemia may be a predictive way of screening rats or potentially humans for their future time-course. A low HCT has been previously associated with a complete elimination of the symptoms of EAE^31^, which is a largely representative condition of the motor neuron spinal cord inflammation seen here. These authors propose that iron deficiency may impair CD4+ T cell maturity, which is also suggested in another study showing human subjects with low iron levels were less responsive to an influenza vaccine^32^. Further, these authors also identify that their mice with a low HCT also have significantly elevated levels of IL-2, which helps promote development of CD25+ regulatory T cells. These regulatory cells may help temper the effects of the CD4+ and CD8+ T lymphocytes, preventing the spinal cord inflammation in the first place. It is unclear why these two rats are anemic to begin with; we do not believe that it has anything to do with the intervention itself, but rather by natural variation. It is possible that iron deficiency and/or HCT may be used clinically to help screen patients for better outcomes in future CNS gene therapy studies, but a better mechanistic understanding of the relationship is critical.

Conversely, tacrolimus rats #3 and #7 both lost expression compared to the other rats in their group. We show that these two rats had normal levels of RDW at 6 weeks compared to the placebo group (P = 0.3), but significantly lower when compared with the other rats in their own group (P = 4E-3). Previous research has shown a correlation between tacrolimus concentrations and RDW in blood samples from anemic transplant recipients^33,34^. This correlation was identified as important for proper titration of tacrolimus dosage in transplant recipients with these authors proposing that RDW could be used to adjust tacrolimus dosage to the patient because “anisocytosis may affect the apparent plasma clearance of tacrolimus”. Our findings suggest the opposite, that tacrolimus is in fact causing the anisocytosis, in which case measures of RDW to predict tacrolimus dosage response may not be appropriate. Rats #3 and #7 may not have absorbed as much tacrolimus as evidenced by a positive immune panel (+serum antibodies, high muscle atrophy, low ChR2 nerve expression, and elevated ipsilateral spinal cord infiltrate) as well as their normal RDW readings. Mechanistically, further study of the cause-effect relationships of immunosuppressants and hematological abnormalities is warranted and may help improve outcomes for both organ transplant and future optogenetic therapy recipients.

In this study, we propose a pharmacological mechanism to extend optogenetic longevity for peripheral nerves. However, it is unclear to what extent the findings in this study are specific to peripheral nerves. Like the BBB, the spinal cord is generally protected from immune attack by the BSCB, which can become dysfunctional in autoimmune conditions like MS^35^. However, we note inflammatory infiltrates directly within the spinal cord that resulted in the irreversible destruction of spinal motor neurons, likely leading to a permanent unilateral weakness in the optogenetic treated rats. Although this destruction of spinal motor neurons may present an opportunity for a new murine model of amyotrophic lateral sclerosis (ALS) based on optogenetics, it is an unnerving finding in the face of planned investigational studies for optogenetics within human peripheral nerves. If we were to inject the virus directly into the spinal cord, would we still identify the same immunogenicity? Given that hSyn properly restricts ChR2 expression to neural tissue and that spinal cord ventral motor neurons project out into the peripheral nervous system, we postulate that immunogenicity is more a function of dosage and cell type than injection location. Using components of the immune panel we developed specifically to screen for optogenetic immunogenicity in this study, we encourage future researchers to evaluate presence of plasma anti-ChR2 antibodies in response to optogenetic transduction directly within the brain and retina to determine whether our results are truly peripheral nerve specific.

Future work should focus on mitigating the immune response by designing opsins to evade immune recognition. One strategy to approach this may be first to identify peptide fragments that are most immunogenic and then alter these regions with site-directed mutagenesis or alternative opsin sequence alignment. Other strategies could focus on the host, either optimizing the immunosuppression drug, dose, and time course or identifying if there is a causative link between anemia and optogenetic immunogenicity, using hematological strategies to evade immune recognition. A third strategy could employ anti-inflammatory constructs such as PD-L1/2 within the AAV DNA to reduce the danger signals required for T lymphocyte mediated cellular destruction^36^. Additionally, this study focused on injection of neonatal and young rats – it would be interesting to identify whether immunosuppression could improve expression in adults, a group more difficult to transduce with AAV. While this study represents an important discovery in optogenetics and neuroimmunology, it is also a humbling one – it underscores the importance of exhaustive safety and validation testing of new technologies prior to their clinical and commercial implementations.

## Methods

All animal experiments were conducted on Fischer 344 or Sprague Dawley rats; all experimental protocols were approved by the Committee on Animal Care at the Massachusetts Institute of Technology. All methods were carried out in accordance with relevant guidelines and regulations.

### Opsin Injection

Sprague Dawley (Horizon Discovery) or Fischer 344 (Charles River Labs) rats were injected at P2 (unless otherwise specified) with 15 µL AAV6-hSyn-ChR2(H134R)-EYFP-WPRE (unless otherwise specified). The use of the hSyn promoter restricted expression to neural tissue^4^. Virus was produced by Virovek, Inc from plasmids at a titer of 1 × 10^14^ vp/mL. Under isoflurane anesthesia, rats were injected under sterile conditions through a 34 G needle (WPI) attached to an intraocular kit (WPI), Silflex tubing (WPI), and a 10 μL nanofil syringe (WPI) on the UMP3 syringe pump (WPI) with injection rate set to 75 nL/s. Rats were housed under a 12:12 light:dark cycle in a temperature-controlled environment with food and water ad libitum, and euthanized at either 8 or 12 weeks of age (unless otherwise specified). Each rat was analyzed for an optogenetic response either weekly or bi-weekly via transdermal illumination to a 105 mW/mm^2^ (unless otherwise specified) 473 nm DPSS laser (OptoEngine) at varying laser intensities and/or a transdermal 0.6 W 475 nm LED (XP-E2, Cree, Inc.). Both laser and LED were set to 5 Hz and 5% DC (unless otherwise specified), illuminating the skin at the proximal tibia transdermal to the peroneal nerve (unless otherwise specified).

### Dosing, anatomy and timing groups

54 Fischer 344 neonatal rats were split into several groups to test the effect of different viral dosages, injection location, and injection timing. To test the effect of dosage on expression levels, six groups of three P2 neonates were injected in the right lower limb – both AC and posterior compartment (PC) – with 15 µL of AAV6-hSyn-ChR2(H134R)-EYFP-WPRE at the following dosages in vp/mL: 3E11, 1E12, 3E12, 1E13, 3E13, 1E14. ChR2(H134R) was chosen due its common usage in the rat peripheral nervous system^3–5^. Two weeks later, the TA on these animals was surgically exposed and rats were injected with another 12 µL directly into the TA and 3 µL directly into the peroneal nerve at the end plate at the same viral concentration, reaching the following total vp injected: (1) 1E10, (2) 3E10, (3) 1E11, (4) 3E11, (5) 1E12, (6) 3E12. To test the effect of injecting in the nerve vs. the muscle, four groups of four rats each were injected with the same virus at 1E14 vp/mL in the following groups: (1) 10 µL in the TA at 2 weeks postpartum; (2) 8 µL in the TA and 2 µL in the peroneal nerve at 2 weeks postpartum; (3) 5 µL in the TA at P2 and 5 µL in the surgically exposed TA 2 weeks later; (4) 5 µL in the TA at P2, 4 µL in the surgically exposed TA and 1 µL in the peroneal nerve at 2 weeks later. To test for the effect of injecting in different ages, four groups of five rats each were injected with the same virus at 1E14 vp/mL in the following groups: (1) 10 µL in the TA at P2; (2) 5 µL in the TA at P2 and 4 µL in the exposed TA & 1 µL in the exposed peroneal nerve at 2 weeks; (3) 2.5 µL in the TA at P2, P6, P10, and P14; (4) 2.5 µL each in the TA at P2 & 1 week postpartum, and 2.0 µL in the exposed TA & 0.5 µL in the exposed peroneal nerve each at 2 weeks postpartum and 3 weeks postpartum. Sample sizes were chosen to identify broad expression patterns without the intent of making statistically significant conclusions between groups. Rats were also exposed at 4 and 6 weeks to direct illumination of the surgically exposed sciatic nerve. Rats were euthanized when transdermal illumination no longer produced any electrophysiological spikes, at a minimum of 8 weeks. This represented 8–12 weeks for all animals with the exception of one animal, which still expressed transdermal up to 72 weeks post-injection (Fig. 1a,b,d).

### Rag2^−/−^ and WT Groups

28 Sprague Dawley neonates were divided into 6 groups. 9 WT and 9 Rag2^−/−^ P2 neonates received injections of 15 µL virus. Of these rats, (1) four WT and (2) four Rag2^−/−^ rats comprised the excitotoxicity control groups. These animals did not receive any light stimulation over the course of the 12 weeks except during terminal procedures. The remaining (3) five WT and (4) five Rag2^−/−^ rats comprised the high-dose group. Lastly, (5) five WT and (6) five Rag2^−/−^ P2 neonates received injections of 1.5 µL virus comprising the low dose group. Rag2^−/−^ rats were housed in SCID caging; liberal use of bleach and Quatricide PV (Pharmacal) prevented infection during testing procedures. Further, all testing was transdermal in these groups, eliminating the need for surgery.

### Drug screen groups

Thirty Sprague Dawley neonates were divided into three groups of 10 neonates each based on the slow-release pellet (Innovative Research of America) employed: Placebo, Tacrolimus (Sigma), and PS2 (Bio-X-Cell). Dosages were sourced from the literature: Tacrolimus: 5.0 mg/kg/day = 30 mg/pellet^37^, and PS2: 0.95 mg/kg/day = 5.5 mg/pellet^29,38^. PS2 was lyophilized using isopropanol in dry ice to freeze liquid followed by 48 hour vacuum to sublimate ice crystals. All drug pellets were produced from powders, manufactured to release drug evenly over a 60-day period based on 100 g animal body weight per pellet using a proprietary matrix (Innovative Research of America). Each group of rats was housed separately. At P2, neonates were injected with 15 µL virus. When rats reached 50 g at roughly 2 weeks of age, a 2–4 mm incision was made ~5 mm caudal to the right ear and each rat was implanted with a single pellet. At ~5 weeks of age, when male rats reached 175 g, and when female rats reached 125 g, an incision was made ~5 mm caudal to the left ear; each male rat was given two additional slow release pellets while each female rat was given only one additional slow release pellet. This accounted for the female weight being ~33% less than the male weight from 5 week up through the point of euthanasia to maintain consistent dosing within a group.

### CAG and No reporter groups

Five Fischer 344 rats were injected with 15 µL AAV6-hSyn-ChR2(H134R)-EYFP-WPRE and five Fisher 344 rats were injected with 15 µL AAV6-CAG-ChR2(H134R)-EYFP-WPRE, at the same 1E14 vp/mL dosage. These animals were tested every two weeks but additionally had one surgery as described previously^3^ at week 4 post-injection to check for optogenetic activity directly on the sciatic nerve. These rats were euthanized at 8 weeks as opposed to 12 weeks. Three of the five CAG promoter rats within two different cages died at ~4 weeks. A necropsy was performed on one of these rats, which revealed no obvious cause of death. Lastly, for the no reporter group, 10 Sprague Dawley rats were injected with 15 µL AAV6-hSyn-ChR2(H134R)-WPRE, eliminating the EYFP construct. Five of these rats were randomly selected to be euthanized at 8 weeks and five of these rats were selected to be euthanized at 12 weeks to increase the chance of catching optogenetic activity within the nerve for immunofluorescence analysis.

### Channelrhodopsin electrophysiology measurement

Laser and LED pulses were controlled using a myDAQ (National Instruments) controlled by the NI Elvis Function Generator and custom software written in MATLAB as described previously^3^. The presence of a foot twitch in response to illumination was evaluated both electrophysiologically and visually. To evaluate the presence of a twitch response electrophysiologically and subsequently measure the strength of that response, four 30 G monopolar electromyography (EMG) needles (Natus Medical) were directly inserted through the skin into the gastrocnemius (GN) and tibialis anterior (TA) muscles of each rat for bipolar recording as described previously^4^. Needles were connected to a 20 kS/s multi-channel amplifier with a fixed 200x gain (IntanTech). The laser and LED were secured above the anesthetized animal to an assembly allowing for six degrees of freedom. The laser (OptoEngine) employed a beam of Gaussian cross-sectional profile and 3 mm diameter (1/e^2^), corresponding to a peak irradiance at the surface of the skin of 105 mW/mm^2^ at a measured output power of 375 mW. Electrical signals controlling the laser amplitude, pulse width, and frequency were simultaneously recorded by the amplifier, enabling temporal synchronization of laser pulses and EMG. EMG data was processed in MATLAB.

### Lysate Production and ChR2-EYFP ELISA

To obtain ChR2-EYFP protein, hippocampal cells from Swiss-Webster mice were cultured in DMEM (D6171, Sigma) containing 1% L-glutamine, 1% Pen-Strep, and 10% Fetal Bovine Serum in a 24 well plate. Cells were transduced with 2 μL AAV6-hSyn-ChR2(H134R)-EYFP-WPRE (Western Blot and ELISA) or 2 μL AAV6-hSyn-ChR2(H134R)-WPRE (Western Blot only) at 1E14 vp/mL (Virovek). After 72–96 hours, EYFP production was assessed with fluorescence microscopy. Wells of successfully expressing plates were washed with 500 μL of sterile ice-cold PBS and incubated with 300 μL of trypsin for 2–4 min at 37 °C. 700 μL of culturing media were added to deactivate the trypsin. Resultant solution was transferred to a conical tube and centrifuged at low speed (650 g) for 5 min at 4 °C. Supernatant was decanted and cell pellet was gently resuspended in 1 ml of ice cold PBS. Solution was centrifuged at low speed (650 g) for 5 min at 4 °C and supernatant was decanted. Cell pellet was resuspended in 400 μL cell lysis buffer (ThermoFisher Scientific) with 40 μL protease inhibitor cocktail (P8340, Sigma), incubated for 30 min on ice, and then centrifuged for 10 min at 12000 RPM at 4 °C. The supernatant was decanted and frozen at −80 °C until use. Western blot was used to verify presence of EYFP (ab290, Abcam) and ChR2 (anti-ChR2, American Research Products) within lysate samples via co-localized bands at ~62 kDa (Supp. Fig. 1a).

To measure serum IgG to ChR2-EYFP, a sandwich capture ELISA was developed on 96 well polystyrene plates (Nunc MaxiSorp, ThermoFisher Scientific). 100 μL/well pAb rabbit anti-GFP (ab290, Abcam) at a 1:500 dilution in PBS was used as the capture Ab and incubated overnight at 4 °C, followed by blocking with 5% skim milk in PBS-T for 1 hour at room temperature in a rocking platform. 40 μL lysate containing ChR2-EYFP was incubated per well for 3 hours at room temperature in a rocking platform. ELISA validation was performed at 1:3 lysate dilution in PBS whereas most sample measurements were performed at 1:20 dilution in PBS. A standard curve of absorbance for a variety of samples at 1:3 and 1:20 lysate dilution showed a linear trend with R^2^ = 0.97 (Supp. Fig. 1b). All reported values in this manuscript have been scaled to the predicted 1:3 lysate dilution.

40 μL detection antibody was added to each well for 2 hours, consisting of either mAb mouse anti-ChR2 (American Research Products, Inc.) diluted at 1:10 in blocking solution for positive controls or rat plasma samples. To identify optimal plasma dilution, samples were tested undiluted, at 1:30, and at 1:900 dilution in PBS suggesting that no dilution gave the greatest sensitivity between test groups (Supp. Fig. 1d). 100 μL HRP-conjugated goat anti-rat or anti-mouse secondary antibodies (ThermoFisher Scientific) diluted at 1:2500 in PBS was incubated per well for 1 hour. Lastly, 100 μL of 0.5 mg/mL OPD (ThermoFisher Scientific) in 90% deionized water and 10% stable peroxide substrate buffer (ThermoFisher Scientific) was added to each well. In between each step, wells were washed 3X for 5 minutes each with 100 μL PBS-T, except for the step prior to OPD, which required 5X washes. Absorbance at 450 nm was measured 15 minutes following the addition of OPD using a SpectraMax M5e plate reader (Molecular Devices). To account for inter-plate variability, each plate was linearly scaled relative to a negative serum control (serum from a non-transduced rat) and a repeated high-expressing serum sample from the same animal.

### Biodistribution

Tissues for the biodistribution study were sourced from the Fischer 344 rats in the 3E12 and 3E10 dosage groups (n = 3 each) as well as the CAG group (n = 2). The tissues investigated included brain, heart, liver, kidney, cervical spinal cord, lungs, ipsilateral axillary lymph node, spleen, gastrocnemius, sciatic nerve, contralateral gastrocnemius, and contralateral sciatic nerve. All tissues were harvested at 8 weeks, snap-frozen and stored in microcentrifuge tubes at −80 °C. 5 mg of tissue was sampled from each collected specimen and DNA extraction from the various organs was performed using DNeasy Tissue Kit (Qiagen) with the addition of RNase to obtain pure DNA. DNA yields were quantified using Qubit Fluorometric Quantitation (ThermoFisher Scientific). Viral DNA was assessed by quantitative PCR (employing an absolute quantification method with a standard curve) on the DNA extract. After primer screening, two sets specific for ChR2 coding sequence were employed: Primer I (HPLC purified): (F) 5′-CAATGTTACTGTGCCGGATG-3′, (R) 5′-ATTTCAATGGCGCACACATA-3′, Primer II: (F) 5′-GCCTACCAAACCTGGAAATCTA-3′, (R) 5′-CTGTGGCAAGGTAGAGCATAG-3′). Samples were retained and used in parallel to analyze both 5 ng (once) and 15 ng (twice) of DNA in triplicates from each tissue. The standard curve was prepared with viral DNA, extracted from the viral particles themselves and purified using the same DNA extraction kit, with 5 mg GN tissue from non-injected control rat for purpose of tissue sample mimicry. Negative controls comprised control (non-injected) rat genomic DNA from the same set of tissues. 40 cycles were run on Roche Light Cycler 480 using SYBR Green I dye chemistry from KAPA SYBR Fast qPCR Master Mix (KAPA BIOSYSTEMS), followed by a melting curve for specificity analysis. The threshold was defined as 66 ChR2 copies per ng of tissue DNA, based on the limit of the dynamic range of the standard curves from all 3 runs. This level is similar to the previously mentioned biodistribution study^25^, which set a threshold of 20 copies of beta actin for every viral copy as a threshold, which corresponds to roughly 20 copies/ng DNA in our study, below the confidence of the standard curve.

### Tissue and sample processing, histology, immunohistochemistry, flow cytometry, and analysis

Blood collection at 6 weeks was done via tail vein draw of ~0.5 mL. Following EMG recordings during terminal procedures, ~0.5 mL blood was collected from rats via cardiac puncture. K3EDTA was used for anticoagulation (Minicollect, Greiner Bio-One). Rats were euthanized via intra-peritoneal sodium barbital followed by transcardial perfusion with 60 mL PBS followed by 60 mL 4% PFA in PBS. Both AC and PC muscle groups on ipsilateral and contralateral legs were carefully dissected, cut from their origin and insertion points, and weighed. Spinal cord, ipsilateral TA, and ipsilateral sciatic nerve were dissected, fixed for 24 hours, paraffin processed, embedded, and cross-sectioned at 10 µm. Spinal cord was sectioned in either axial or coronal orientation.

Complete blood counts (CBCs) were performed with automated differential (Hemavet 950SS, Drew Scientific). For flow cytometery samples, blood was diluted by 1X in PBS and then spun down (650 g, 15 min) over Lymphoprep (Stem Cell Technologies). White blood cells predominantly comprising peripheral blood mononuclear cells (PBMCs) were collected from phase layer, spun down again in PBS (650 g, 5 min). Supernatant was decanted and 500 µL PBS containing 1% BSA was added to resuspend PBMCs followed by 1.25 µL PE-conjugated Ms mAb to CD3 (ab95509, Abcam) or 1.6 µL isotype control (ab172730, Abcam). After a 30-minute incubation at 4 °C, samples were spun once again, supernatant was discarded, and 100 µL PBS containing 1% BSA was added to each sample followed by resuspension. Samples were incubated overnight at 4 °C. Flow Cytometry (BD FACSCelesta) was used to identify CD3+ lymphocytes. For non-flow cytometry samples, blood was spun down (650 g, 15 min), plasma was collected and frozen at −80 °C.

For immunofluorescence and immunohistochemistry, EYFP expression was amplified with Rb pAb anti-GFP (ab290, Abcam) at 1:200 or Gt pAb anti-GFP (ab6673, Abcam) at 1:100 with either anti-Rb or anti-Gt Alexa Fluor 488 (Fisher) or anti-Rb or anti-Gt AP (Biocare) and Vulcan Fast Red (Biocare). Expression of ChR2 was amplified with Ms anti-ChR2 at 1:50 (American Research Products, Inc.) and anti-Ms Alexa Fluor 488 (Fisher). Immunohistochemistry and immunofluorescence of inflammatory infiltrates employed Ms mAb anti-CD8α (ab33786, Abcam) or Ms mAb anti-CD68 (ab31630, Abcam) with either anti-Ms Alexa Fluor 568 (Fisher) or Goat anti-Ms HRP (GHP516, Biocare) with DAB (DB801, Biocare). All antibodies were diluted in 1% w/v BSA in PBS-T. Immunofluorescence images were taken on an Evos FL Auto epifluorescence microscope (Fisher) at 10x (spinal cord) or 20x (sciatic nerve). H&E and immunohistochemistry images were taken on a digital slide scanner (Aperio AT2, Leica) at 20x. Using ImageJ, opsin^+^ axons were counted manually whereas total axon counts were estimated from representative counts of subsets of the nerve. Assessment of spinal cord inflammation was performed on ImageJ 10X DAPI+ sections using the freehand selection tool to choose equivalent areas of ventral horn gray matter on left and right coronal lumbar sections. The ImageJ process employed inversion, thresholding, conversion to masks, watershedding, and the “analyze particles” function with size limits set to 30–250 square pixels per cell. The number of total particles (corresponding roughly to the number of cells) was scaled to the total measured area of each selection.

### Statistical analysis

Statistical significance was calculated in Microsoft Excel with the data analysis toolbox. For comparisons of individual groups, student’s two-tailed t-tests with unequal variance were performed. For comparisons of multiple groups, a single factor analysis of variance (ANOVA) was performed followed by post hoc Fisher’s Least Significant Difference (LSD) test for significance. All data represent the mean ± s.d. of at least three independent experiments unless otherwise specified; the number of trials is reported in the data provided.

### Code availability

The MATLAB.m code used for the temperature simulation, EMG processing and MC simulation are available from the corresponding author upon reasonable request.

## Electronic supplementary material

Supplementary Information

Supplementary Video

## Data Availability

The authors declare that all data supporting the findings of this study are available within the manuscript and its supplementary information.
