# Supplementary material for: Optogenetic Peripheral Nerve Immunogenicity
Source: Sci Rep. 2018 Sep 19;8:14076. doi: 10.1038/s41598-018-32075-0 (PMC6145901; doi:10.1038/s41598-018-32075-0)
Supplement: Supplementary file 1 — Supplementary Information [file 41598_2018_32075_MOESM1_ESM.pdf]

Optogenetic Peripheral Nerve Immunogenicity

Authors: Benjamin E. Maimon[1,2], Maurizio Diaz[3], Emilie C. M. Revol[4], Alexis M. Schneider[5], Ben Leaker[2], Claudia E. Varela[2], Shriya Srinivasan[1,2], Matthew B. Weber[1,6], and Hugh M. Herr[1,*]

[1]MIT Media Lab, Center for Extreme Bionics, Massachusetts Institute of Technology, Cambridge, MA, USA
[2]Harvard-MIT Division of Health Sciences and Technology (HST), Massachusetts Institute of Technology, Cambridge, MA, USA
[3]Department of Electrical Engineering and Computer Science, Massachusetts Institute of Technology, Cambridge, MA, USA
[4]Department of Bioengineering, École Polytechnique Fédérale de Lausanne (EPFL), Lausanne, Switzerland
[5]Department of Biological Engineering, Massachusetts Institute of Technology, Cambridge, MA, USA
[6]Harvard-MIT Division of Health Sciences and Technology (HST), Harvard Medical School, Boston, MA, USA

Lead Contact: Benjamin Maimon. E-mail: bmaimon@media.mit.edu
[*]Corresponding author: Hugh M. Herr, MIT Media Lab, Center for Extreme Bionics, Massachusetts Institute of Technology, Cambridge, MA, USA, Tel: (617) 258-6574, E-mail: hherr@media.mit.edu

Table of Contents

**Supplementary Video 1**	72 week transdermal optogenetic stimulation, CAG promoter muscle stimulation, and hSyn promoter stimulation with & without EYFP reporter
**Supplementary Figure 1**	ELISA validation testing
**Supplementary Figure 2**	$Rag2^{-/-}$ and WT inflammation comparison
**Supplementary Figure 3**	Tacrolimus and placebo inflammation comparison
**Supplementary Figure 4**	PS2 mAb Results
**Supplementary Figure 5**	CAG Biodistribution and ELISA
**Supplementary Figure 6**	Excitotoxicity control results
**Supplementary Figure 7**	With and without fluorescent reporter inflammation comparison

Supplementary Video 1: Video outlining concepts described in the manuscript, split into three sections. The first (0:07) shows transdermal optogenetic activation of motor activity in right hindlimb of rat 72 weeks post-injection using blue (470 nm) LEDs. The second (0:15) shows transdermal optogenetic activation of rat right hindlimb with CAG promoter showing direct muscle activation and inversion/eversion control. The third (0:36) shows transdermal optogenetic activation of rat right hindlimb with hSyn promoter both with and without EYFP promoter.

Supplementary Figures

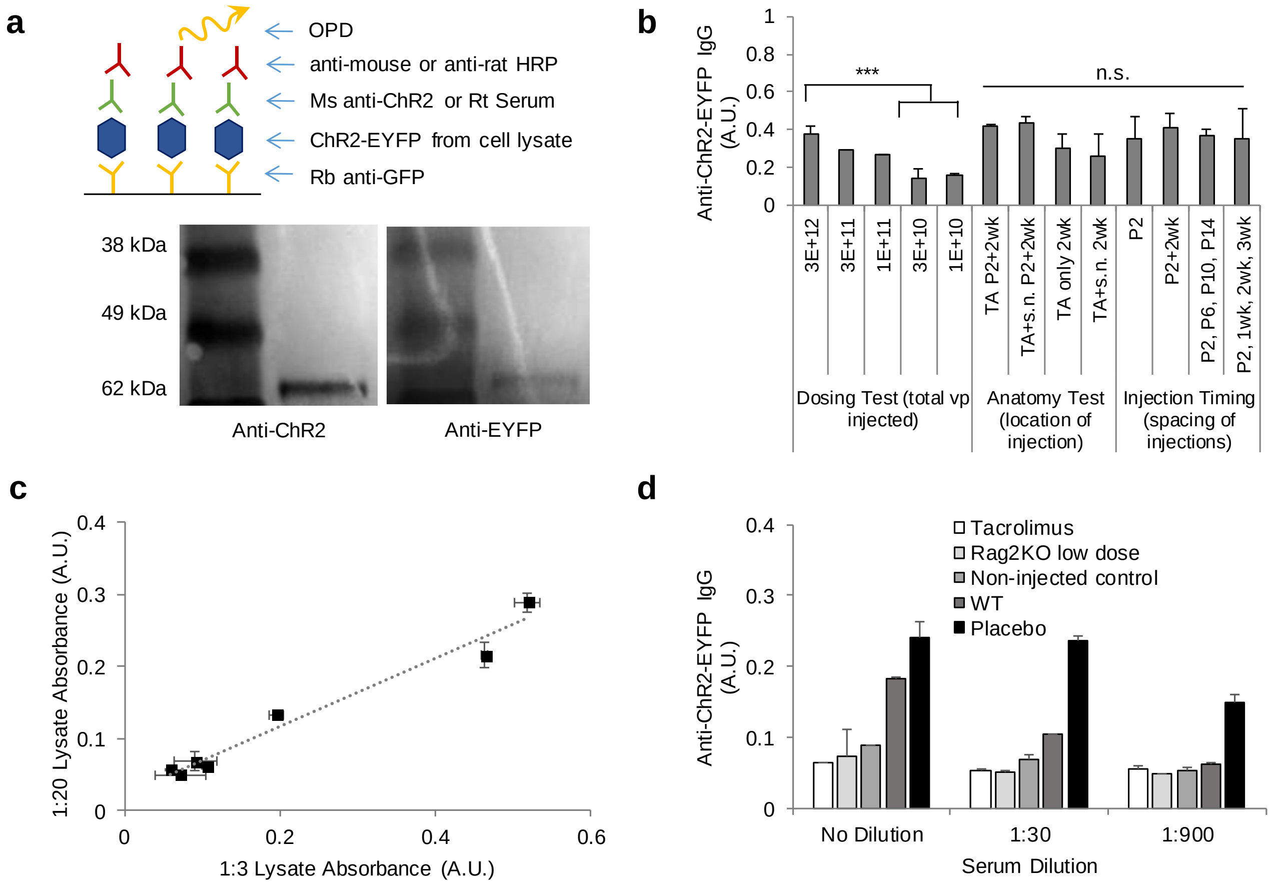

Supplementary Figure 1: ELISA validation testing. (a) Schematic of sandwich capture ELISA for detection of serum antibodies to ChR2-EYFP and western blot of cell lysate showing co-localization with both ChR2 & EYFP specific primary antibodies. Images are from two different gels, stained with two different primary antibodies. (b) Lysate dilution validation test for the ELISA protocol. There is a linear relationship between absorbance measured with lysate diluted 1:3 in PBS and lysate diluted 1:20 in PBS ($R^2 = 0.97$). This result was used to scale all ELISA measurements to the expected value at 1:3 lysate dilution. (c) Terminal ELISA results for the dosage, anatomy, and timing rats showing a significant difference in anti-ChR2-EYFP antibody production between the 1E12 and 1E10 AAV dosage groups (P = 7E-4). No significant difference within the anatomy and timing groups (P_{ANOVA} = .34). (d) Serum dilution validation test for the ELISA protocol. Serums were tested at no dilution, 1:30 dilution in PBS, and 1:900 dilution in PBS (n = 2 each).

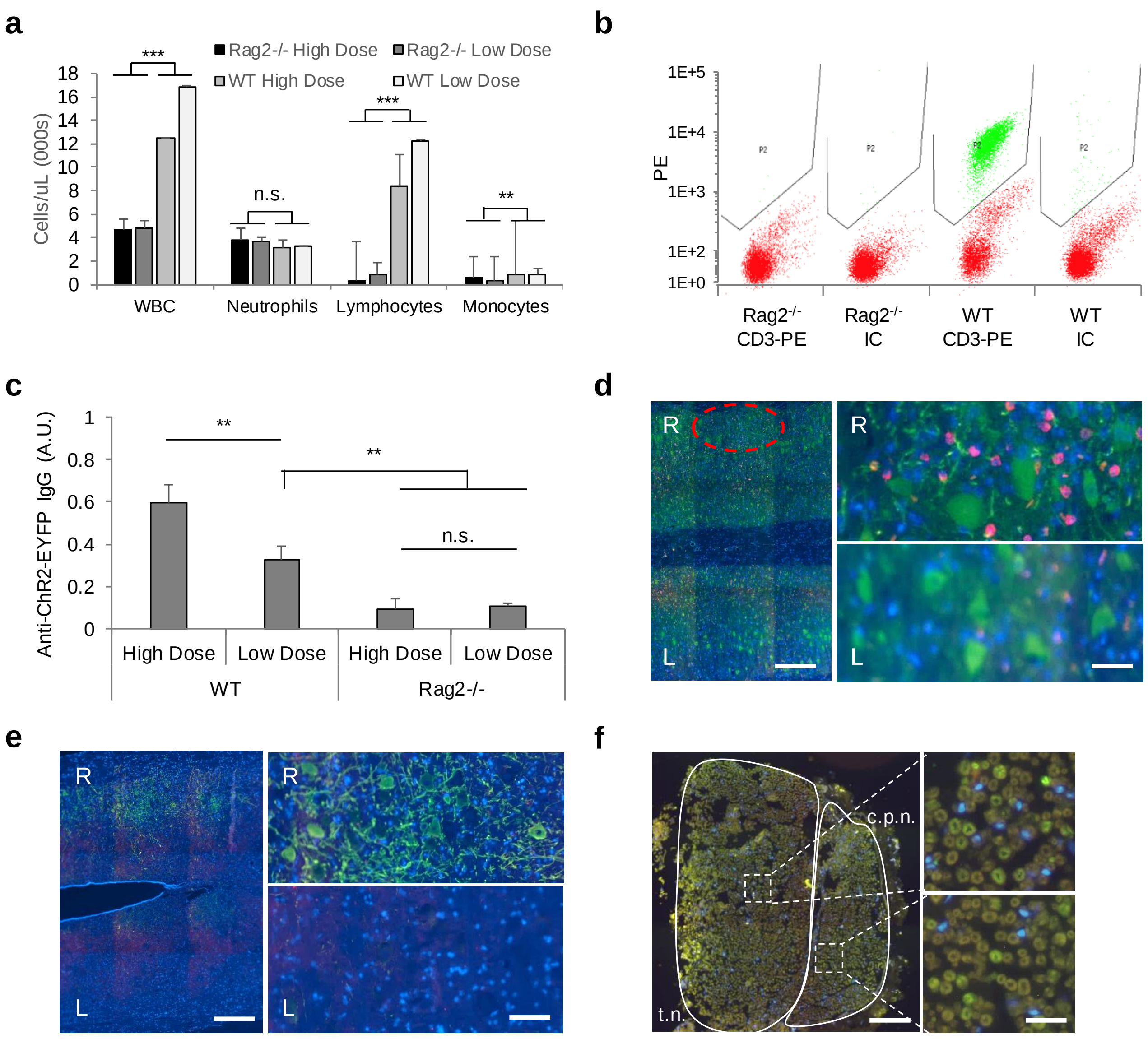

Supplementary Figure 2: Rag2$^{-/-}$ and WT inflammation comparison. (a) White blood cell (WBC) counts for Rag2$^{-/-}$ and WT rats at low and high AAV6 injection doses 6 weeks post-injection with auto-differential ($n_{Rag2-/- \text{ High Dose}}$ = 3, $n_{Rag2-/- \text{ Low Dose}}$ = 4, $n_{WT \text{ High Dose}}$ = 5, $n_{WT \text{ Low Dose}}$ = 2). P_{WBC} = 2E-4, $P_{Neutrophils}$ = 0.2, $P_{Lymphocytes}$ = 2E-4, $P_{Monocytes}$ = 8E-3. (b) Flow cytometry for both Rag2$^{-/-}$ and WT rat peripheral blood mononuclear cells (PBMCs) stained for either CD3-Phycoerythrin (PE) or Isotype Control (IC). (c) Normalized ELISA comparing plasma antibodies against ChR2(H134R)-EYFP for Rag2$^{-/-}$ (n = 5 each) and WT (n=4 each) rats at high vp dose (1.5E12 vp) and low vp dose (1.5E11 vp) at 12 weeks post injection (P_{left} = .003, P_{right} = .003). The one WT high-dose animal which still expressed transdermal at 12 weeks was excluded. (d) Coronal spinal cord section from WT rat stained for nuclei (DAPI, blue), neurons (green), and CD8 (red). Red oval circles neuron-poor region containing many CD8+ cells (right): Scale bar (left) = 500 µm. Scale bar (right) = 70 µm. (e) Coronal spinal cord section from Rag2$^{-/-}$ rat stained for nuclei (DAPI, blue), ChR2-EYFP (green), and CD8 (red). Scale bar (left) = 500 µm. Scale bar (right) = 120 µm. (f) Nerve cross-section from WT rat #1 which maintained expression along with ChR2+ axon counts in tibial nerve (t.n.) division and common peroneal nerve (c.p.n.): Scale bar (left) = 120 µm. Scale bar (right) = 20 µm.

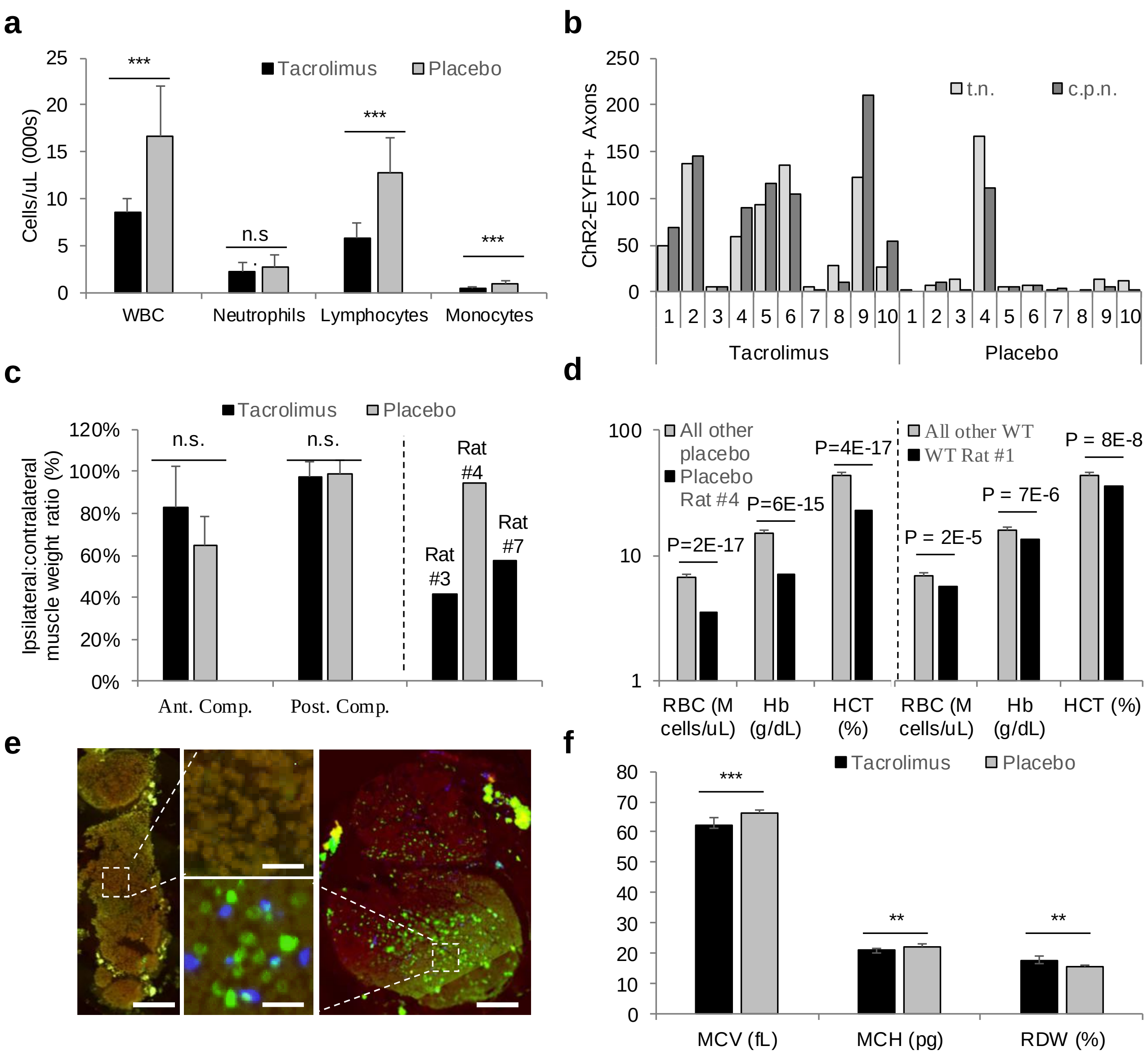

Supplementary Figure 3: Tacrolimus and placebo inflammation comparison. (a) White blood count (WBC) results for tacrolimus and placebo rats at 6 weeks post-injection with auto-differential ($n_{Tacrolimus}$ = 10, $n_{Placebo}$ = 10). P_{WBC} = 7E-4, $P_{Neutrophils}$ = 0.2, $P_{Lymphocytes}$ = 1E-4, $P_{Monocytes}$ = 2E-4. (b) Complete counts of ChR2-EYFP+ axons in tibial nerve (t.n.) and peroneal nerve (c.p.n.) for tacrolimus and placebo groups. (c) Ipsilateral to contralateral side of injection muscle weight ratio at the time of euthanasia between tacrolimus and placebo treated rats for both anterior (AC) and posterior compartment (PC) muscle groups (n=10 per group). P_{AC} = .09 and P_{PC} = .37. The muscle weight ratio for rats #3 and #7 in the tacrolimus group, which had lost expression at week 12, and rat #4 in the placebo group, which maintained expression at week 12, are shown separately as well as included in the totals. (d) Red Blood Cell (RBC), hemoglobin (Hb), and hematocrit (HCT) counts for both placebo and wild-type (WT) rats along with outliers, which maintained long-term expression shown separately. (e) Immunofluorescence from outlier tacrolimus rat #3 (left) and placebo rat #4 (right) with zoom-in sections for each. Scale bar = 200 μm (left, right). Scale bar = 20 μm (center). (f) Mean Corpuscular Volume (MCV), Mean Corpuscular Hemoglobin (MCH), and Red Blood Cell Distribution Width (RDW) for tacrolimus and placebo treated animals suggesting tacrolimus-induced iron deficiency anemia (low MCV and high RDW). P_{MCV} = 9E-4, P_{MCH} = 4E-3, P_{RDW} = 1E-3.

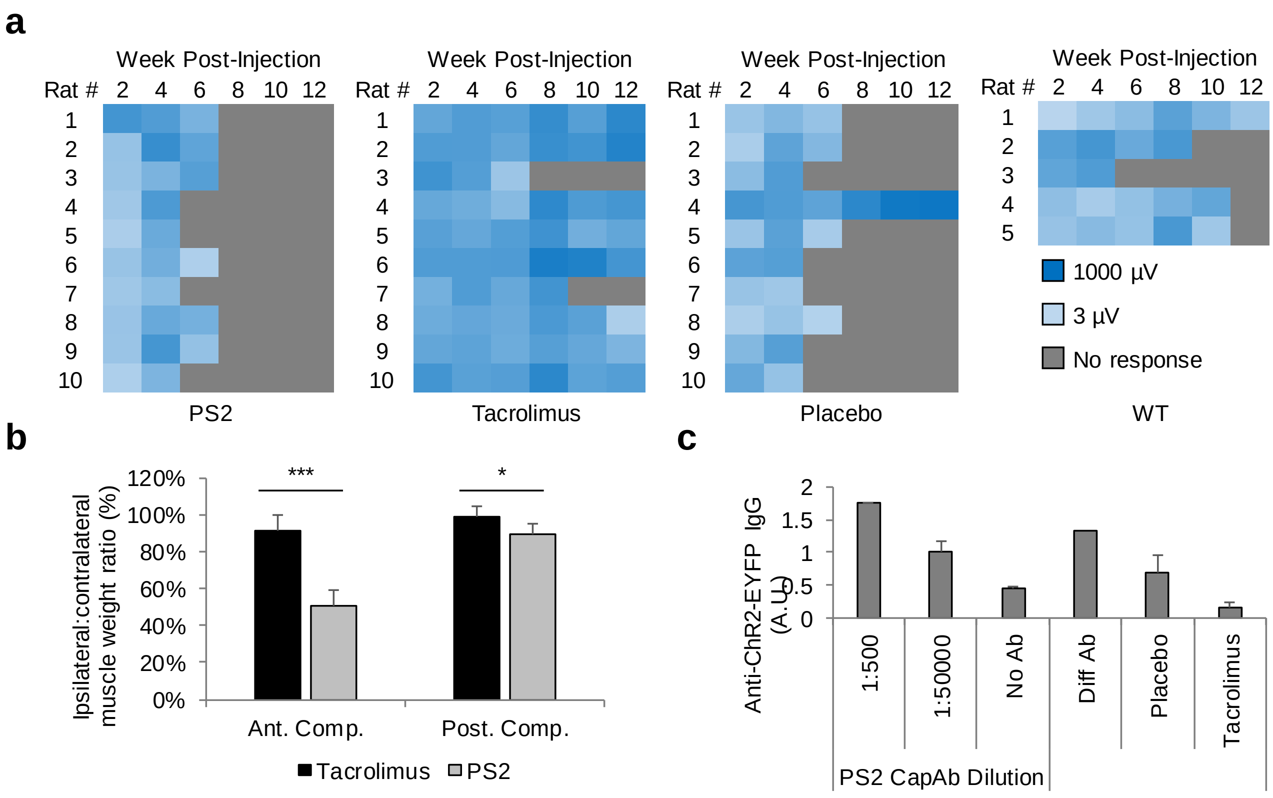

Supplementary Figure 4: PS2 mAb Results. (a) Logarithmic V_{RMS} amplitude of Tibialis Anterior (TA) motor activity in response to 473 nm, 105 mW/mm^2 transdermal illumination of the proximal tibia for 4 s at 5 Hz and 10 ms PW for rats treated with PS2, tacrolimus, placebo, and nothing (WT). (b) Ipsilateral to contralateral side of injection muscle weight ratio at the time of euthanasia between PS2 and Tacrolimus rats for both the anterior and posterior compartment muscle groups (n_{PS2}=10, $n_{Tacrolimus}$=8). Tacrolimus rats #3 and #7 (which had lost expression at time of euthanasia) were excluded. $P_{AntComp.}$ = 4E-7 and $P_{PostComp}$ = .02. (c) Normalized ELISA showing development of neutralizing rat anti-antibody antibodies in PS2 population. Plasma antibodies against for PS2 rat treated with unique concentrations of capture antibody as well as same rat with a Rb anti-GAP43 antibody without any lysate (Diff Ab) along with placebo and tacrolimus are shown for comparison. Signal is much higher for PS2 rats in absence of lysate compared to placebo and tacrolimus rats indicating non-specific binding of rat antibodies to any capture antibody on the ELISA plate.

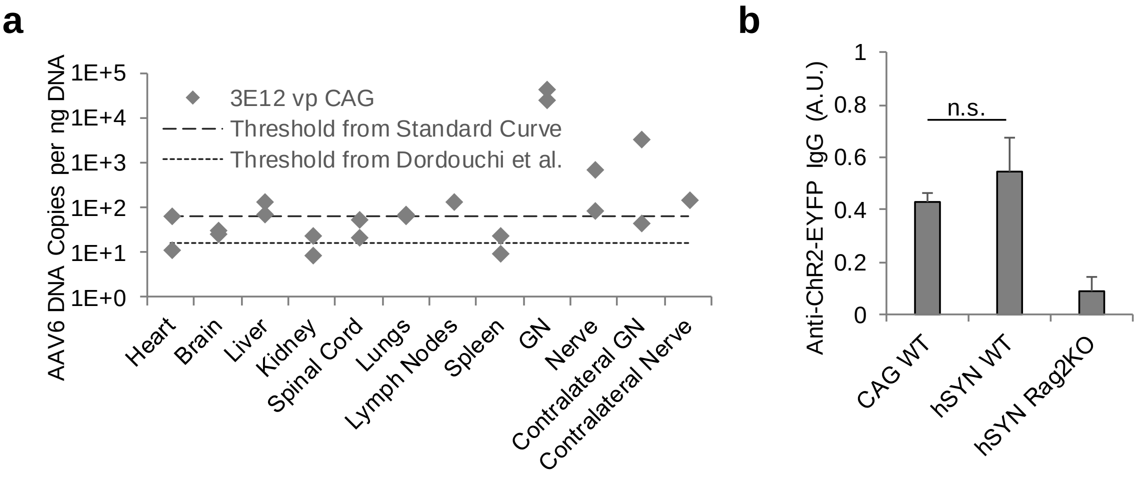

Supplementary Figure 5: CAG Biodistribution and ELISA. (a) Biodistribution results for 3E12 vp AAV6-CAG-ChR2-EYFP injected rats. (GN = Gastrocnemius Muscle, Nerve = Sciatic Nerve), n = 2. (b) Normalized ELISA comparing plasma antibodies against ChR2(H134R)-EYFP for transduction using CAG promoter and hSyn promoter 12 weeks post injection (n = 2 for CAG WT, n = 5 for hSyn WT, n = 5 for hSyn Rag2$^{-/-}$).

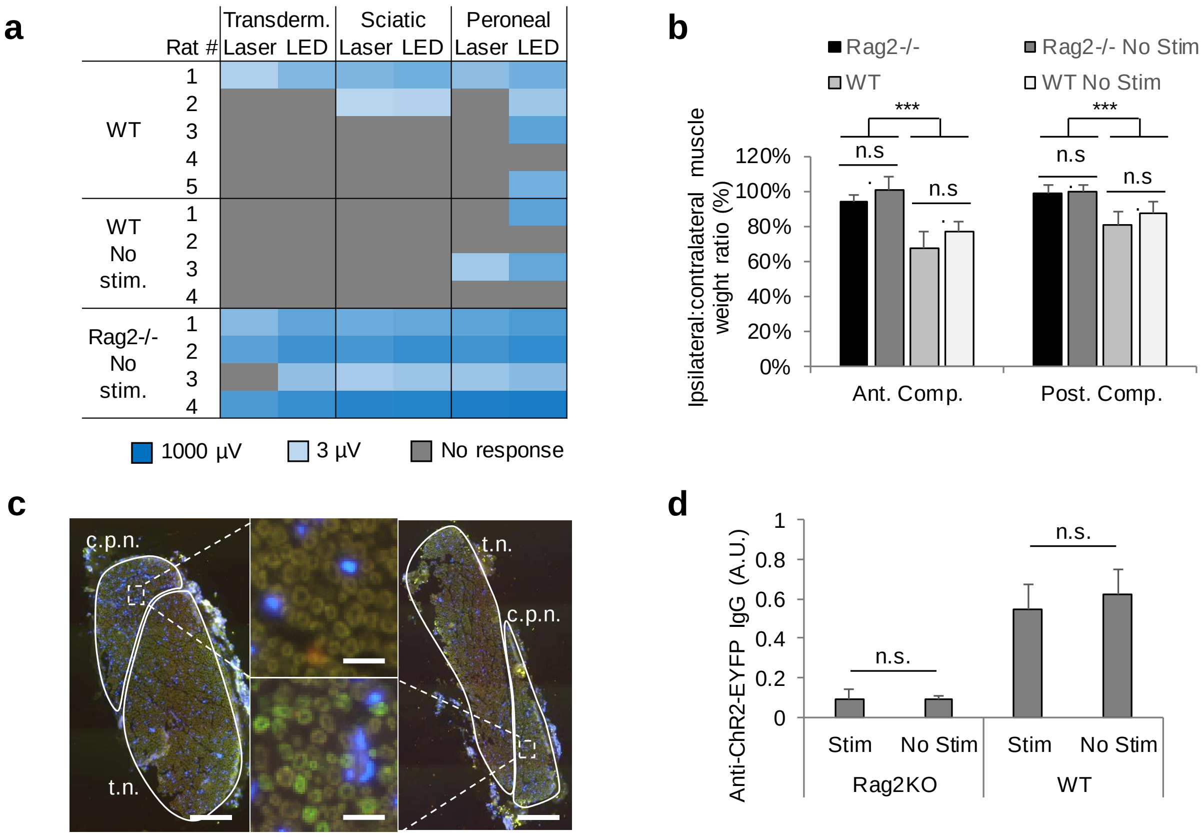

Supplementary Figure 6: Excitotoxicity control results. (a) Logarithmic V_{RMS} amplitude of Tibialis Anterior (TA) motor activity in response to 473 nm, 105 mW/mm^2 laser and 600 mW LED illumination of the proximal tibia (transdermal), the surgically exposed sciatic nerve (sciatic) and the surgically exposed peroneal nerve (peroneal) during terminal procedures. Illumination comprised 4 s at 5 Hz and 10 ms PW for both light sources. WT animals have been illuminated transdermal every 2 weeks from injection, whereas WT and Rag2$^{-/-}$ no stim are being illuminated for the first time during terminal procedures at 12 weeks. (b) Ipsilateral to contralateral side of injection muscle weight ratio at the time of euthanasia between Rag2$^{-/-}$ (n = 5), Rag2$^{-/-}$ no stim (n = 4), WT (n = 5), and WT no stim (n = 4) rats for both anterior (AC) and posterior (PC) compartment muscle groups. P_{Rag2AC} = .14, P_{WTAC} = .10, P_{Rag2PC} = .30, P_{WTPC} = .21, $P_{Rag2WTAC}$ = 8E-6, $P_{Rag2WTPC}$ = 2E-4. (c) Sciatic nerve from WT (left) and Rag2$^{-/-}$ (right) no stimulation controls showing no ChR2-EYFP+ axons and many ChR2-EYFP+ axons respectively: scale bar = 150 µm and 20 µm. (d) Normalized ELISA comparing plasma antibodies against ChR2(H134R)-EYFP for Rag2$^{-/-}$ and WT animals, with and without transdermal stimulation 12 weeks post injection (Rag2$^{-/-}$: n = 5 for Stim group, n = 3 for No Stim group; WT: n = 5 for Stim group, n = 3 for No Stim Group). Note: WT no stim. rat #3 excluded from ELISA because blood not properly collected.

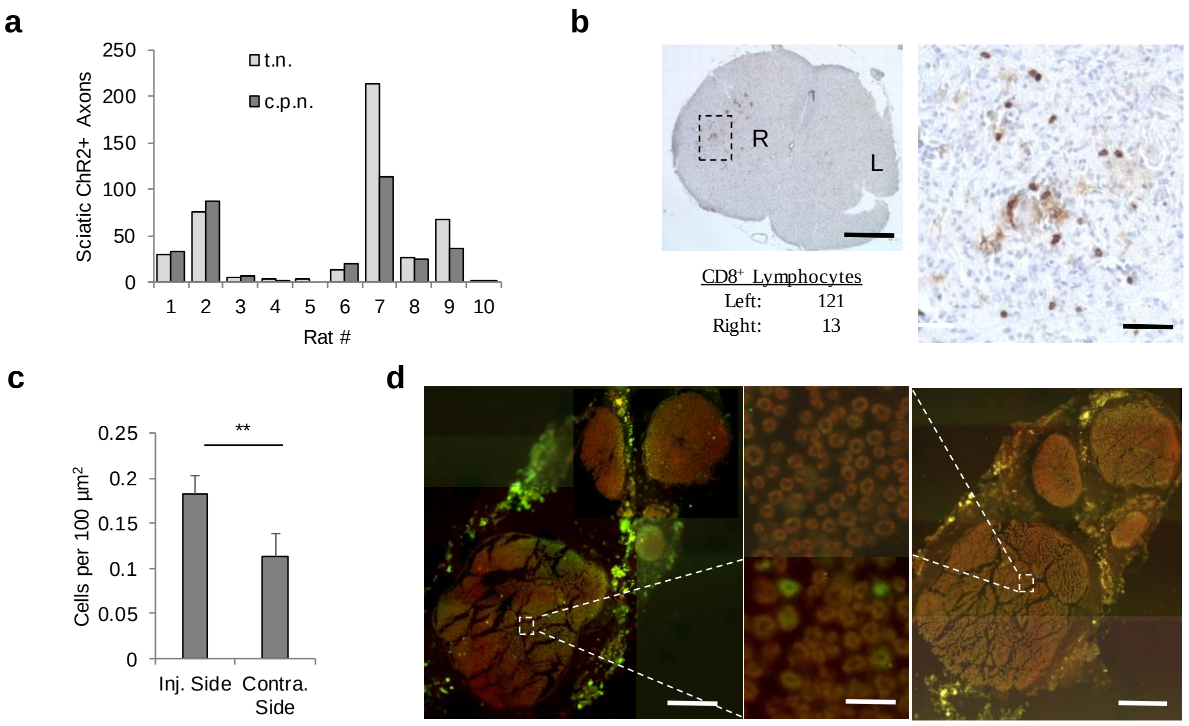

Supplementary Figure 7: With and without fluorescent reporter inflammation comparison. (a) ChR2+ Axons as a percentage of total axons in tibial nerve (t.n.) and peroneal nerve (c.p.n.) for no reporter animals. No reporter rat #7 had maintained transdermal expression at time of euthanasia. (b) Spinal cord cross-section of rat #2 showing CD8+ lymphocytes (brown) on ipsilateral side of Tibialis Anterior injection: scale bar (left) = 1 mm. scale bar (right) = 20 µm. (c) Number of cells per 100 square microns within ventral horn gray matter (n=3) using Student's T test paired samples (P = .005). (d) No reporter sciatic nerve stained for anti-ChR2 (green, left) and for anti-GFP (green, right) along with zoom-in sections for each shown: scale bar (left, right) = 150 µm; scale bar (center) = 15 µm.
